# A Study of Impulsive Multiterm Fractional Differential Equations with Single and Multiple Base Points and Applications

**DOI:** 10.1155/2014/194346

**Published:** 2014-01-21

**Authors:** Yuji Liu, Bashir Ahmad

**Affiliations:** ^1^Department of Mathematics, Guangdong University of Business Studies, Guangzhou 510320, China; ^2^Department of Mathematics, Faculty of Science, King Abdulaziz University, P.O. Box 80203, Jeddah 21589, Saudi Arabia

## Abstract

We discuss the existence and uniqueness of solutions for initial value problems of nonlinear singular multiterm impulsive Caputo type fractional differential equations on the half line. Our study includes the cases for a single base point fractional differential equation as well as multiple base points fractional differential equation. The asymptotic behavior of solutions for the problems is also investigated. We demonstrate the utility of our work by applying the main results to fractional-order logistic models.

## 1. Introduction

The theory of impulsive differential equations describes processes which experience a sudden change of their state at certain moments. Processes with such a characteristic arise naturally and are often, for example, studied in physics, chemical technology, population dynamics, biotechnology, and economics. These processes are modeled by impulsive differential equations. In 1960, Milman and Myshkis introduced the concept of impulsive differential equations [1]. Afterwards, this subject was extensively investigated and several monographs have been published by many authors like Samoilenko and Perestyuk [2], Lakshmikantham et al. [3], Baino and Simeonov [4], Baino and Covachev [5], and Benchohra et al. [6].

Fractional differential equations (FDEs for short), regarded as the generalizations of ordinary differential equations to an arbitrary noninteger order, find their genesis in the work of Newton and Leibniz in the seventieth century. Recent investigations indicate that many physical systems can be modeled more accurately with the help of fractional derivatives [7]. Fractional differential equations, therefore, find numerous applications in the field of viscoelasticity, feedback amplifiers, electrical circuits, electroanalytical chemistry, fractional multipoles, and neuron modelling encompassing different branches of physics, chemistry, and biological sciences [8–10].

Some recent work on the existence of solutions for initial value problems of Caputo type impulsive fractional differential equations can be found in a series of papers [11–16], whereas the solvability of boundary value problems of impulsive differential equations involving Caputo fractional derivatives was investigated in [17–26].

In the left and right fractional derivatives *D*_*a* ^+^_^*α*^ *x* and *D*_*b* ^−^_^*α*^ *x*, *a* is called a left base point and *b* right base point. Both *a* and *b* are called base points of fractional derivatives. A fractional differential equation (FDE) containing more than one base points is called a *multiple base points FDE* while an FDE containing only one base point is called a *single base point FDE*.

Henderson and Ouahab [12] studied the solvability of the following initial value problems for impulsive fractional differential equations:

(1) $$\begin{array}{c} D_{*}^{\alpha }u\left(t\right)=f\left(t,u\left(t\right)\right),\;t\in \left(0,b\right]\setminus \left\{t_{1},\ldots ,t_{m}\right\},\;\;\alpha \in \left(1,2\right],\\ u\left(t_{k}^{+}\right)=I_{k}\left(u\left(t_{k}^{-}\right)\right),\;k=1,2,\ldots ,m,\\ u^{\prime }\left(t_{k}^{+}\right)=J_{k}\left(u\left(t_{k}^{-}\right)\right),\;k=1,2,\ldots ,m,\\ u\left(0\right)=a,\;\;u^{\prime }\left(0\right)=c,\\ D_{*}^{\alpha }u\left(t\right)=f\left(t,u\left(t\right)\right),\;t\in \left(0,b\right]\setminus \left\{t_{1},\ldots ,t_{m}\right\},\;\;\alpha \in \left(0,1\right],\\ \Delta u\left(t_{k}\right)=u\left(t_{k}^{+}\right)-u\left(t_{k}^{-}\right)=I_{k}\left(y\left(t_{k}^{-}\right)\right),\;k=1,2,\ldots ,m,\\ u\left(0\right)=a, \end{array}$$

where 0 < *t*_1_ < *t*_2_ < ⋯<*t*_*m*_ < *b*, *b* > 0 is a fixed real number, *f* : [0, *b*] × *R* → *R* is continuous, *I*_*k*_, *J*_*k*_ : *R* → *R*  (*k* = 1,2,…, *m*) are continuous functions, *u*(*t*_*k*_^+^) = lim⁡_*t*→*t*_*k*_^+^_⁡*u*(*t*) and *u*(*t*_*k*_^−^) = lim⁡_*t*→*t*_*k*_^−^_⁡*u*(*t*) and *u*′(*t*_*k*_^+^) = lim⁡_*t*→*t*_*k*_^+^_⁡*u*′(*t*). One can see that both fractional differential equations in (1) are multiple base points FDEs with base points 0, *t*_1_, *t*_2_,…, *t*_*m*_, which are in fact the impulse points.

In [27], the authors used the concept of upper and lower solutions together with Schauder's fixed point theorem to study the impulsive fractional-order differential equation:

(2) Dcαu(t)=f(t,u(t)), t∈(0,b]∖{t1,…,tm},  α∈(0,1],Δu(tk)=u(tk+)−u(tk−)=Ik(y(tk−)), k=1,2,…,m,u(0)=a.

One can notice that the problem (2) contains a multiple base points FDE with base points 0, *t*_1_, *t*_2_,…, *t*_*m*_ (impulse points).

In [28], the authors studied the existence and uniqueness of solutions of the following initial value problem of fractional order differential equations:

(3) Dcαu(t)=f(t,u(t)), t∈(0,b]∖{t1,…,tm},  α∈(1,2],Δu(tk+)=Ik(u(tk−)), k=1,2,…,m,Δu′(tk)=Jk(u(tk−)), k=1,2,…,m,u(0)=u0,  u′(0)=u1,

where the fractional differential equations are a multiple base points FDE with the base points 0, *t*_1_, *t*_2_,…, *t*_*m*_ (impulse points).

Fečkan et al. [29] studied the existence of solutions of the following initial value problem of impulsive fractional differential equations:

(4) $$\begin{array}{c} D_{0^{+}}^{\alpha }u\left(t\right)=f\left(t,u\left(t\right)\right),\;t\in \left(0,b\right]\setminus \left\{t_{1},\ldots ,t_{m}\right\},\;\;\alpha \in \left(0,1\right],\\ u\left(t_{k}^{+}\right)=I_{k}\left(y\left(t_{k}^{-}\right)\right),\;k=1,2,\ldots ,m,\\ u\left(0\right)=a, \end{array}$$

where 0 < *t*_1_ < *t*_2_ < ⋯<*t*_*m*_ < *b*, *b* > 0 is a fixed real number, *f* : [0, *b*] × *R* → *R* is jointly continuous, *I*_*k*_ : *R* → *R*  (*k* = 1,2,…, *m*) are continuous functions, *u*(*t*_*k*_^+^) = lim⁡_*t*→*t*_*k*_^+^_⁡*u*(*t*) and *u*(*t*_*k*_^−^) = lim⁡_*t*→*t*_*k*_^−^_⁡*u*(*t*) and *u*′(*t*_*k*_^+^) = lim⁡_*t*→*t*_*k*_^+^_⁡*u*′(*t*). Observe that the fractional differential equation in (4) is a single base point FDE with the base point *t* = 0. So the impulse points are different from the base point.

Liu and Ahmad [30] studied a problem of multi-term and multiorder quasi-Laplacian singular fractional differential equations:

(5) D0+β[Φ(ρ(t)D0+αx(t))]  +q(t)f(t,x(t),D0+αx(t))=0, t∈(0,+∞),lim⁡t→0t1−αx(t)=∫0+∞m(t)g(t,x(t),D0+αx(t))dt,lim⁡t→+∞I0+1−βΦ(ρ(t)D0+αx(t))  =∫0+∞n(t)h(t,x(t),D0+αx(t))dt,Δx(tk)=Ik(tk,x(tk),D0+αx(tk)), k=1,2,…,ΔΦ(ρ(tk)D0+αx(tk))=Jk(tk,x(tk),D0+αx(tk)),                      k=1,2,…,

where 1 < *α*,  *β* ≤ 1,  0 < *t*_1_ < *t*_2_ < ⋯ are fixed points, *D*_0^+^_ is the Riemann-Liouville fractional derivative, Φ : *R* → *R* is a sup-multiplicative function, *f*, *g*, *h* are impulsive Caratheodory functions, *m*, *q*, *n*, *ρ* : (0,1)→(0, +*∞*) are continuous functions, and *I*_*k*_, *J*_*k*_ are impulse functions. In (5), the fractional differential equation is a single base point FDE with the base point *t* = 0. Clearly the impulse points are different from the base point.

**Remark**

It is clear from the abovementioned work that IVPs of impulsive fractional differential equations can be categorized into two classes: (a) IVPs of one base point FDEs [20, 29, 30] and (b) IVPs of multiple base points FDEs [12, 27, 28].

In this paper, we study the following two initial value problems (IVPs for short) of nonlinear multi-term FDEs with impulses on half lines:

(6) cD0+αx(t)=q(t)f(t,x(t),Dc0+px(t)), t∈(0,∞),x(0)=x0,Δx(tk)=Ik(tk,x(tk)), k=1,2,…,

(7) cD∗αx(t)=q(t)f(t,x(t),Dc∗px(t)), t∈(0,∞),x(0)=x0,Δx(tk)=Ik(tk,x(tk)), k=1,2,…,

where *x*_0_ ∈ *R*, *α* ∈ (0,1], 0 < *p* < *α*, 0 = *t*_0_ < *t*_1_ < *t*_2_ < *t*_3_ < ⋯ with lim⁡_*k*→*∞*_⁡*t*_*k*_ = *∞*, ^*c*^ *D*_0^+^_ is the standard Caputo fractional derivative at the base point *t* = 0, *q* : (0, *∞*) → *R* satisfies that there exists *l* > −*α* such that |*q*(*t*)|≤*t* ^*l*^ for all *t* ∈ (0, *∞*), *q* may be singular at *t* = 0, ^*c*^ *D*_∗_ is the standard Caputo fractional derivative at the base points *t* = *t*_*k*_  (*k* = 1,2,…); that is, ^*c*^ *D*_∗_^*α*^|_(*t*_*k*_,*t*_*k*+1_]_ *u*(*t*) =  ^*c*^ *D*_*t*_*k*_^+^_^*α*^ *u*(*t*) for all *t* ∈ (*t*_*k*_, *t*_*k*+1_], and *f* : [0, *∞*) × *R* ^2^ → *R* is a Caratheodory function, *I*_*k*_ : (0, *∞*) × *R* → *R*  (*k* = 1,2,…) and {*I*_*k*_} is a Caratheodory function sequence, and Δ*x*(*t*_*k*_) = lim⁡_*t*→*t*_*k*_^+^_⁡*x*(*t*) − lim⁡_*t*→*t*_*k*_^−^_⁡*x*(*t*),  *k* = 1,2,….

The salient features of the present work include the following: (i) to establish sufficient conditions for the existence of solutions for the IVP (6) with a single base point and IVP (7) with multiple base points (same as the impulse points). We emphasize that the conditions for the existence of solutions for the IVPs (6) and (7) are different; (ii) the asymptotic behavior of solutions for the problems is studied and the sufficient criterion for every solution to tend to zero as *t* → *∞* is established; (iii) the method of proof relies on the Schauder fixed point theorem; (iv) our approach for dealing with impulsive problems at hand is different from the ones employed in earlier work on the topic and thus opens a new avenue for studying impulsive fractional differential equations; (v) as an application, we apply our results to fractional-order logistic models and present sufficient conditions for the existence and asymptotic behavior of solutions of these logistic models.

The paper is organized as follows: the auxiliary material is given in Section 2, the main results are presented in Sections 3 and 4, while the application of the main results is demonstrated in Section 5.

## 2. Preliminaries

We recall some basic concepts of fractional calculus [9, 10] and show auxiliary results.

Define the Gamma function and Beta function, respectively, as

(8) Γ(α1)=∫0+∞sα1−1e−sds,B(α2,β2)=∫01(1−x)α2−1xβ2−1dx,          α1>0, α2,β2>0.

**Definition 1 (see [9])**

Riemann-Liouville fractional integral of order *α* > 0 of a continuous function *f* : (0, *∞*) → *R* is given by

(9) $$\begin{array}{c} I_{0+}^{\alpha }f\left(t\right)=\frac{1}{\Gamma \left(\alpha \right)}\int_{0}^{t}{\left(t-s\right)}^{\alpha -1}f\left(s\right)ds, \end{array}$$

provided that the right-hand side exists.

**Definition (see [9])**

Caputo's derivative of fractional-order *α* for a function *f* ∈ *AC* ^(*n*−1)^([0, *∞*), *R*) is defined by

(10) $$\begin{array}{c} D_{0^{+}}^{\alpha }f\left(t\right)=\frac{1}{\Gamma \left(n-\alpha \right)}\int_{0}^{t}{\left(t-s\right)}^{n-\alpha -1}f^{\left(n\right)}\left(s\right)ds, \end{array}$$

for *n* − 1 < *α* ≤ *n*, *n* ∈ *N*. If 0 < *α* ≤ 1, then

(11) $$\begin{array}{c} D_{0^{+}}^{\alpha }f\left(t\right)=\frac{1}{\Gamma \left(1-\alpha \right)}\int_{0}^{t}{\left(t-s\right)}^{-\alpha }f^{\left(1\right)}\left(s\right)ds. \end{array}$$

Obviously, Caputo's derivative of a constant is zero.

**Lemma 3 (see [9])**

For *α* > 0, the general solution of fractional differential equation ^*c*^ *D*_0^+^_^*α*^ *x*(*t*) = 0 is given by *x*(*t*) = *c*_0_ + *c*_1_ *t* + *c*_2_ *t* ^2^ + ⋯+*c*_*n*−1_ *t* ^*n*−1^, where *c*_*i*_ ∈ *R*, *i* = 0,1, 2,…, *n* − 1,  *n* − 1 < *α* ≤ *n*.

**Definition**

A function *x* : [0, *∞*) → *R* is said to be a solution of the IVP (6) if both *x*|_(*t*_*k*_,*t*_*k*+1_]_  (*k* = 0,1, 2,3,…) and ^*c*^ *D*_0^+^_^*p*^ *x*|_(*t*_*k*_,*t*_*k*+1_]_  (*k* = 0,1, 2,3,…) are continuous, *x* satisfies the differential equation ^*c*^ *D*_0^+^_^*α*^ *x*(*t*) = *q*(*t*)*f*(*t*, *x*(*t*), ^*c*^ *D*_0^+^_^*p*^ *x*(*t*)) a.e. on (0, *∞*)∖{*t*_1_, *t*_2_, *t*_3_,…}, and the limits lim⁡_*t*→*t*_*k*_^+^_⁡*x*(*t*) and lim⁡_*t*→*t*_*k*_^+^_⁡^*c*^ *D*_0^+^_^*p*^ *x*(*t*)  (*k* = 0,1, 2,3,…) exist and the following conditions are satisfied:

(12) $$\begin{array}{c} \Delta x\left(t_{k}\right)=I_{k}\left(t_{k},x\left(t_{k}\right)\right),\;k=1,2,\ldots ,\;\;x\left(0\right)=x_{0}. \end{array}$$

**Definition 5**

A function *x* : [0, *∞*) → *R* is said to be a solution of the IVP (7) if both *x*|_(*t*_*k*_,*t*_*k*+1_]_  (*k* = 0,1, 2,3,…) and ^*c*^ *D*_0^+^_^*p*^ *x*|_(*t*_*k*_,*t*_*k*+1_]_  (*k* = 0,1, 2,3,…) are continuous, *x* satisfies the differential equation ^*c*^ *D*_*t*_*k*_^+^_^*α*^ *x*(*t*) = *q*(*t*)*f*(*t*, *x*(*t*), ^*c*^ *D*_*t*_*k*_^+^_^*p*^ *x*(*t*)) on (*t*_*k*_, *t*_*k*+1_], and the limits lim⁡_*t*→*t*_*k*_^+^_⁡*x*(*t*) and lim⁡_*t*→*t*_*k*_^+^_⁡^*c*^ *D*_0^+^_^*p*^ *x*(*t*)  (*k* = 0,1, 2,3,…) exist and the following conditions are satisfied:

(13) $$\begin{array}{c} \Delta x\left(t_{k}\right)=I_{k}\left(t_{k},x\left(t_{k}\right)\right),\;k=1,2,\ldots ,\;\;x\left(0\right)=x_{0}. \end{array}$$

Choose *σ* > max⁡{0, *α* + *l*} and *μ* > max⁡{*σ*, *σ* − *α* − *l*}. Let

(14) X={x:[0,∞)⟶R:x|(tk,tk+1]∈C0(tk,tk+1], k=0,1,2,…,Dc0+px|(tk,tk+1]∈C0(tk,tk+1], k=0,1,2,…,tσ−α−l(1+t)(1+tμ)x(t)  is  bounded  on⁡  (0,∞)tp+σ−α−l1+tμDc0+px(t)  is  bounded  on⁡  (0,∞).}.

For *x* ∈ *X*, define the norm on *X* as

(15) ||x||=max⁡{sup⁡t∈(0,∞)tσ−α−l(1+t)(1+tμ)|x(t)|,    sup⁡t∈(0,∞)tp+σ−α−l1+tμ|Dc0+px(t)|}.

It is easy to show that *X* is a real Banach space.

**Definition**

*f* : [0, +*∞*) × *R* ^2^ → *R* is called a Caratheodory function if it satisfies the following assumptions:

(i) (*t*, *x*, *y*) → *f*(*t*, ((1 + *t*)(1 + *t* ^*μ*^)/*t* ^*σ*−*α*−*l*^)*x*, ((1 + *t* ^*μ*^)/*t* ^*p*+*σ*−*α*−*l*^)*y*) is continuous on [0, +*∞*) × *R* ^2^;
(ii) for each *r* > 0, there exists a constant *M*_*r*_ > 0 such that |*x* | , |*y* | ≤*r* implies that
(16) $$\begin{array}{c} \left|f\left(t,\frac{\left(1+t\right)\left(1+t^{\mu }\right)}{t^{\sigma -\alpha -l}}x,\frac{1+t^{\mu }}{t^{p+\sigma -\alpha -l}}y\right)\right|\leq M_{r},\;t\in \left[0,\infty \right). \end{array}$$

**Definition 7**

{*I*_*k*_} is called a Caratheodory function sequence if it satisfies the following assumptions:

(i) *x* → *I*_*k*_(*t*_*k*_, ((1 + *t*_*k*_)(1 + *t*_*k*_^*μ*^)/*t*_*k*_^*σ*−*α*−*l*^)*x*) is continuous on *R* for each *k* = 1,2, 3,…;
(ii) for each *r* > 0, there exist constants *M*_*rk*_ > 0 such that |*x* | ≤*r* implies that
(17) $$\begin{array}{c} \left|I_{k}\left(t_{k},\frac{\left(1+t_{k}\right)\left(1+t_{k}^{\mu }\right)}{t_{k}^{\sigma -\alpha -l}}x\right)\right|\leq M_{rk},\;\sum_{k=1}^{\infty }M_{rk}<\infty . \end{array}$$

If *b* > *a* > 0, then we have

(18) $$\begin{array}{c} \underset{t\in (0,\infty )}{\sup}\frac{t^{a}}{1+t^{b}}=\frac{1}{b}a^{a/b}{\left(b-a\right)}^{\left(b-a\right)/b}=:M_{a,b}. \end{array}$$

**Lemma 8**

Suppose that *f* is a Caratheodory function and {*I*_*k*_} is a Caratheodory function sequence on *X*. Then *x* ∈ *X* is a solution of

(19) Dc0+αx(t)=q(t)f(t,x(t),Dc0+px(t)), t∈(0,∞),x(0)=x0,Δx(tk)=Ik(tk,x(tk)), k=1,2,…,

if and only if *x* ∈ *X* is a solution of the fractional integral equation

(20) x(t)=x0+∫0t(t−s)α−1Γ(α)q(s)        ×f(s,x(s),Dc0+px(s))ds   +∑j=1kIj(tj,x(tj)), t∈(tk,tk+1],  k=0,1,2,….

**Proof**

For *x* ∈ *X* and *r* > 0, we have

(21) max⁡{sup⁡t∈(0,∞)tσ−α−l(1+t)(1+tμ)|x(t)|,    sup⁡t∈(0,∞)tp+σ−α−l1+tμ|Dc0+px(t)|}=r.

Since *f* is a Caratheodory function and {*I*_*k*_} is a Caratheodory function sequence, therefore, there exist *M*_*r*_ > 0 and *M*_*rk*_ > 0 such that

(22) |f(t,x(t),Dc0+px(t))| =|f(t,(1+t)(1+tμ)tσ−α−ltσ−α−l(1+t)(1+tμ)x(t),     1+tμtp+σ−α−ltp+σ−α−l1+tμ cD0+px(t))| ≤Mr, t∈[0,∞),|Ik(tk,x(tk))| =|Ik(tk,(1+tk)(1+tkμ)tkσ−α−l     ×tkσ−α−l(1+tk)(1+tkμ)x(tk))| ≤Mrk, k=1,2,3,…,  ∑k=1∞Mrk<∞.

Let us assume that *x* satisfies (48). Then, by Lemma 3, the solution of (48) can be written as

(23) x(t)=∫0t(t−s)α−1Γ(α)q(s)f(s,x(s), cD0+px(s))ds+ck,            t∈(tk,tk+1], k=0,1,2,….

Observe that

(24) |∫0t(t−s)α−1Γ(α)q(s)f(s,x(s),Dc0+px(s))ds| ≤Mr∫0t(t−s)α−1Γ(α)slds =Mrtα+l∫01(1−w)α−1Γ(α)dw⟶0 as  t⟶0.

From *x*(0) = *x*_0_ and Δ*y*(*t*_*k*_) = *I*_*k*_(*t*_*k*_, *x*(*t*_*k*_)), we get *c*_0_ = *x*_0_ and

(25) ∫0tk(tk−s)α−1Γ(α)q(s)f(s,x(s),Dc0+px(s))ds+ck  −(∫0tk(tk−s)α−1Γ(α)q(s)      ×f(s,x(s),Dc0+px(s))ds+ck−1) =Ik(tk,x(tk)).

This implies that

(26) ck=ck−1+Ik(tk,x(tk))=x0+∑j=1kIj(tj,x(tj)), k=0,1,2,….

Thus, we have

(27) x(t)=∫0t(t−s)α−1Γ(α)q(s)f(s,x(s),Dc0+px(s))ds+x0+∑j=1kIj(tj,x(tj)).

Hence, *x* satisfies (49). Next, we show that *x* ∈ *X*. Indeed

(28)   cD0+px(t)=∫0t(t−s)α−p−1Γ(α−p)q(s)f(s,x(s),Dc0+px(s))ds.

It is easy to see that

(29) x|(tk,tk+1]∈C0(tk,tk+1], cD0+px|(tk,tk+1]∈C0(tk,tk+1],        k=0,1,2,….

Furthermore, for *t* ∈ (*t*_*k*_, *t*_*k*+1_], we have

(30) tσ−α−l(1+t)(1+tμ)|x(t)| ≤tσ−α−l1+tμ|∫0t(t−s)α−1Γ(α)q(s)f(s,x(s),Dc0+px(s))ds       +x0+∑j=1kIj(tj,x(tj))| ≤tσ−α−l(1+t)(1+tμ)  ×∫0t(t−s)α−1Γ(α)|q(s)f(s,x(s),Dc0+px(s))|ds  +tσ−α−l(1+t)(1+tμ)|x0|  +tσ−α−l(1+t)(1+tμ)∑j=1k|Ij(tj,x(tj))| ≤tσ−α−l(1+t)(1+tμ)∫0t(t−s)α−1Γ(α)Mrslds  +Mσ−α−l,μ|x0|+Mσ−α−l,μ∑j=1kMrk =Mrtσ1+t∫01(1−w)α−1Γ(α)wldw+|x0|+∑j=1kMrk ≤MrMσ,μB(α,l+1)Γ(α)+Mσ−α−l,μ|x0|  +Mσ−α−l,μ∑j=1∞Mrk<∞,tσ+p−α−l1+tμ|Dc0+px(t)| =tσ+p−α−l1+tμ  ×|∫0t(t−s)α−p−1Γ(α−p)q(s)f(s,x(s),Dc0+px(s))ds| ≤tσ+p−α−l1+tμ∫0t(t−s)α−p−1Γ(α−p)Mrslds =Mrtσ1+tμ∫01(1−w)α−p−1Γ(α−p)wldw ≤MrMσ,μB(α−p,l+1)Γ(α−p)<∞.

This implies that *x* ∈ *X*. Conversely, suppose that *x* satisfies (49). By a direct computation, it follows that the solution given by (49) satisfies the problem (48). This completes the proof.

Choose *σ* > max⁡{0, *α* + *l*} and *μ* > max⁡{*σ*, *σ* − *α* − *l*} and define

(31) Y={x:[0,∞)⟶R:x|(tk,tk+1]∈C0(tk,tk+1], k=0,1,2,…,Dctk+px|(tk,tk+1]∈C0(tk,tk+1], k=0,1,2,…,tσ−α−l(1+t)(1+tμ)x(t)  is  bounded  on⁡  (0,∞){sup⁡t∈(tk,tk+1]⁡tp+σ−α−l1+tμ|Dctk+px(t)|:k=0,1,2,…}  is  bounded.}.

For *x* ∈ *Y*, we define the norm on *Y* as

(32) ||x||=max⁡{sup⁡t∈(0,∞)tσ−α−l(1+t)(1+tμ)|x(t)|,     sup⁡k=0,1,2,…{sup⁡t∈(tk,tk+1]tp+σ−α−l1+tμ|Dctk+px(t)|}}.

It is easy to show that *Y* is a real Banach space.

**Lemma 9**

Suppose that *f* is a Caratheodory function and {*I*_*k*_} is a Caratheodory function sequence, *x* ∈ *Y* and *λ*_0_ = :inf⁡_*k*=0,1,2,…_⁡(*t*_*k*_ − *t*_*k*−1_) > 0. Then *x* ∈ *Y* is a solution of the problem

(33) Dc∗αy(t)=q(t)f(t,x(t),Dc∗px(t)), t∈(0,∞),y(0)=x0,Δy(tk)=Ik(tk,x(tk)), k=1,2,…,

if and only if *x* ∈ *Y* is a solution of the fractional integral equation

(34) x(t)=∫tkt(t−s)α−1Γ(α)q(s)f(s,x(s),Dctk+px(s))ds +x0+∑j=1kIj(tj,x(tj))+∑j=1k∫tj−1tj(tj−s)α−1Γ(α)q(s)f(s,x(s),Dctj−1+px(s))ds,              t∈(tk,tk+1], k=0,1,2,….

**Proof**

For *x* ∈ *Y*, we have that there exists *r* > 0 such that

(35) max⁡{sup⁡t∈(0,∞)tσ−α−l(1+t)(1+tμ)|x(t)|,     sup⁡n=0,1,2,… sup⁡t∈(tk,tk+1]tp+σ−α−l(1+t)(1+tμ)|Dctk+px(t)|}=r.

Since *f* is a Caratheodory function and {*I*_*k*_} is a Caratheodory function sequence, then there exist *M*_*r*_ > 0 and *M*_*rk*_ > 0 such that

(36) |f(t,x(t),Dctk+px(t))|  =|f(t,(1+t)(1+tμ)tσ−α−ltσ−α−l(1+t)(1+tμ)x(t),      1+tμtp+σ−α−ltp+σ−α−l1+tμ cDtk+px(t))|  ≤Mr, t∈[0,∞),|Ik(tk,x(tk))|  =|Ik(tk,(1+tk)(1+tkμ)tkσ−α−l      ×tkσ−α−l(1+tk)(1+tkμ)x(tk))|  ≤Mrk, k=1,2,3,…,  ∑k=1∞Mrk<∞.

Assume that *x* satisfies the problem (50). Then, in view of Lemma 3, we can write the solution of (50) as

(37) x(t)=∫tkt(t−s)α−1Γ(α)q(s)f(s,x(s), cDtk+px(s))ds+ck,         t∈(tk,tk+1], k=0,1,2,….

From *x*(0) = *x*_0_, we get *c*_0_ = *x*_0_. Since

(38) |∫tkt(t−s)α−1Γ(α)q(s)f(s,x(s),Dctk+px(s))ds| ≤Mr∫tkt(t−s)α−1Γ(α)slds =Mrtα+l∫tk/t1(1−w)α−1Γ(α)wldw⟶0       as  t⟶tk+, k=1,2,3,…

and Δ*y*(*t*_*k*_) = *I*_*k*_(*t*_*k*_, *x*(*t*_*k*_)), we get

(39) ck−(∫tk−1tk(tk−s)α−1Γ(α)q(s)f(s,x(s),Dctk−1+px(s))ds    +ck−1)=Ik(tk,x(tk)),

which gives

(40) ck=ck−1+Ik(tk,x(tk))+∫tk−1tk(tk−s)α−1Γ(α)q(s)f(s,x(s),Dctk−1+px(s))ds=x0+∑j=1kIj(tj,x(tj))+∑j=1k∫tj−1tj(tj−s)α−1Γ(α)q(s)f(s,x(s),Dctj−1+px(s))ds,                    k=0,1,2,….

Hence the solution of the problem (50) is

(41) x(t)=∫tkt(t−s)α−1Γ(α)q(s)f(s,x(s),Dctk+px(s))ds+x0+∑j=1kIj(tj,x(tj))+∑j=1k∫tj−1tj(tj−s)α−1Γ(α)q(s)f(s,x(s),Dctj−1+px(s))ds,            t∈(tk,tk+1], k=0,1,2,….

Next, we need to show that *x* ∈ *Y*. Clearly,

(42)   cDtk+px(t)=∫0t(t−s)α−p−1Γ(α−p)q(s)f(s,x(s),Dctk+px(s))ds,x|(tk,tk+1]∈C0(tk,tk+1],Dctk+px|(tk,tk+1]∈C0(tk,tk+1],         k=0,1,2,….

Furthermore, for *t* ∈ (*t*_*k*_, *t*_*k*+1_], we have

(43) tσ−α−l(1+t)(1+tμ)|x(t)| =tσ−α−l(1+t)(1+tμ)  ×|∫tkt(t−s)α−1Γ(α)q(s)f(s,x(s), cDtk+px(s))ds    +x0+∑j=1kIj(tj,x(tj))    +∑j=1k∫tj−1tj(tj−s)α−1Γ(α)q(s)        ×f(s,x(s),Dctj−1+px(s))ds| ≤tσ−α−l(1+t)(1+tμ)∫tkt(t−s)α−1Γ(α)Mrslds  +Mσ−α−l,μ|x0|+Mσ−α−l,μ∑j=1kMrk  +tσ−α−l(1+t)(1+tμ)∑j=1k∫tj−1tj(tj−s)α−1Γ(α)Mrslds ≤Mrtσ(1+t)(1+tμ)∫tk/t1(1−w)α−1Γ(α)wldw  +Mσ−α−l,μ|x0|+Mσ−α−l,μ∑j=1∞Mrk  +tσ−α−l(1+t)(1+tμ)Mr  ×∑j=1ktjα+l∫tj−1/tj1(1−w)α−1Γ(α)wldw ≤MrMσ,μ∫01(1−w)α−1Γ(α)wldw  +Mσ−α−l,μ|x0|+Mσ−α−l,μ∑j=1∞Mrk  +Mr∑j=1ktσ−α−ltjα+l(1+t)(1+tμ)  ×∫tj−1/tj1(1−w)α−1Γ(α)wldw ≤MrMσ,μB(α,l+1)Γ(α)+Mσ−α−l,μ|x0|  +Mσ−α−l,μ∑j=1∞Mrk+Mr∑j=1ktσ−α−ltjα+ltμ+1  ×∫01(1−w)α−1Γ(α)wldw ≤MrMσ,μB(α,l+1)Γ(α)+Mσ−α−l,μ|x0|  +Mσ−α−l,μ∑j=1∞Mrk  +Mr∑j=1ktjα+ltjμ+1−σ+α+lB(α,l+1)Γ(α) ≤MrMσ,μB(α,l+1)Γ(α)+Mσ−α−l,μ|x0|  +Mσ−α−l,μ∑j=1∞Mrk  +Mr∑j=1k1tjμ+1−σB(α,l+1)Γ(α).

Since *t*_*j*_ − *t*_*j*−1_ ≥ *λ*_0_ and *t*_0_ = 0, we get *t*_*j*_ ≥ *jλ*_0_ for all *j* = 0,1, 2,…. Then

(44) tσ−α−l(1+t)(1+tμ)|x(t)| ≤MrMσ,μB(α,l+1)Γ(α)+Mσ−α−l,μ|x0|  +Mσ−α−l,μ∑j=1∞Mrk  +Mr1λ0μ+1−σB(α,l+1)Γ(α)∑j=1∞1jμ+1−σ,    t∈(tk,tk+1], k=0,1,2,….

So

(45) $$\begin{array}{c} \frac{t^{\sigma -\alpha -l}}{\left(1+t\right)\left(1+t^{\mu }\right)}\left|x\left(t\right)\right|\;\;\text{is}\;\;\text{bounded}\;\;\mathrm{on}\;\;\left(0,\infty \right). \end{array}$$

Moreover, for *t* ∈ (*t*_*k*_, *t*_*k*+1_], we get

(46) tp+σ−α−l1+tμ|Dctk+px(t)| =tp+σ−α−l1+tμ|∫tkt(t−s)α−p−1Γ(α−p)q(s)         ×f(s,x(s),Dctk+px(s))ds| ≤tp+σ−α−l1+tμ∫tkt(t−s)α−p−1Γ(α−p)Mrslds =Mrtσ1+tμ∫tk/t1(1−w)α−p−1Γ(α−p)wldw ≤MrMσ,μB(α−p,l+1)Γ(α−p)<∞,     t∈(tk,tk+1], k=0,1,2,….

So

(47) tp+σ−α−l1+tμ|Dctk+py(t)|  is  bounded  on⁡  (0,∞).

Thus, *x* ∈ *Y*. Conversely, assume that *x* satisfies (51). Then, by direct computation, it follows that the solution given by (51) satisfies the problem (50). This completes the proof.

## 3. Existence Results for an IVP with a Single Base Point 

In this section, we discuss the existence and uniqueness of solutions for the single base point IVP (6). The asymptotic behaviour of solutions of IVP (6) is also investigated.

In relation to the IVP (6), we define an operator *T* : *X* → *X* by

(48) (Tx)(t)=∫0t(t−s)α−1Γ(α)q(s) ×f(s,x(s),cD0+px(s))ds+x0+∑j=1kIj(tj,x(tj)),   t∈(tk,tk+1], k=0,1,2,….

**Lemma 10**

Let *f* be a Caratheodory function and let {*I*_*k*_} be a Caratheodory function sequence. Then (i)  *T* : *X* → *X* is well defined; (ii)  the fixed point of the operator *T* coincides with the solution of IVP (6); (iii)  *T* : *X* → *X* is completely continuous.

**Proof**

(i) For *x* ∈ *X*, let

(49) r=max⁡{sup⁡t∈(0,∞)tσ−α−l(1+t)(1+tμ)|x(t)|,    sup⁡t∈(0,∞)tp+σ−α−l1+tμ|Dc0+px(t)|}<+∞.

Since *f* is a Caratheodory function, {*I*_*k*_} is Caratheodory function sequence; there exist positive numbers *M*_*r*_ > 0 and *M*_*rk*_ > 0  (*k* = 1,2,…) such that

(50) |f(t,x(t),Dc0+px(t))|≤Mr, t∈[0,∞),

(51) $$\begin{array}{c} |I_{k}\left(t_{k},x\left(t_{k}\right)\right)|\leq M_{rk},\;k=1,2,\ldots ,\;\;\sum_{k=1}^{\infty }M_{rk}<\infty . \end{array}$$

It is easy to show that

(52) Tx∈C0(tk,tk+1],   cD0+pTx∈C0(tk,tk+1],                  k=0,1,2,….

As in the proof of Lemma 8, it can be shown that both (*t* ^*σ*−*α*−*l*^/(1 + *t*)(1 + *t* ^*μ*^))(*Tx*)(*t*) and  (*t* ^*p*+*σ*−*α*−*l*^/(1 + *t* ^*μ*^)) ^*c*^ *D*_0^+^_^*p*^(*Tx*)(*t*)  are  bounded  on  (0, *∞*).

Hence, *Tx* ∈ *X* and consequently *T* : *X* → *X* is well defined.

(ii) It follows from Lemma 8 that the fixed point of the operator *T* coincides with the solution of IVP (6).

(iii) To establish that *T* is completely continuous, we show that (a) *T* is continuous, (b) *T* maps bounded sets of *X* to bounded sets, and (c) *T* maps bounded sets of *X* to relatively compact sets.

(a) In order to show that the operator *T* is continuous, let *x*_*n*_ ∈ *X* with *x*_*n*_ → *x*_0_ as *n* → *∞*. We will prove that *Tx*_*n*_ → *Tx*_0_ as *n* → *∞*. It is easy to see that there exists *r* > 0 such that


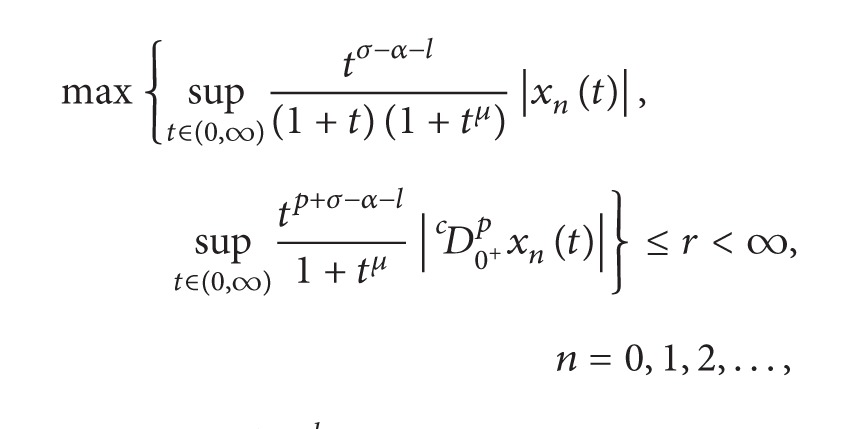

(53)



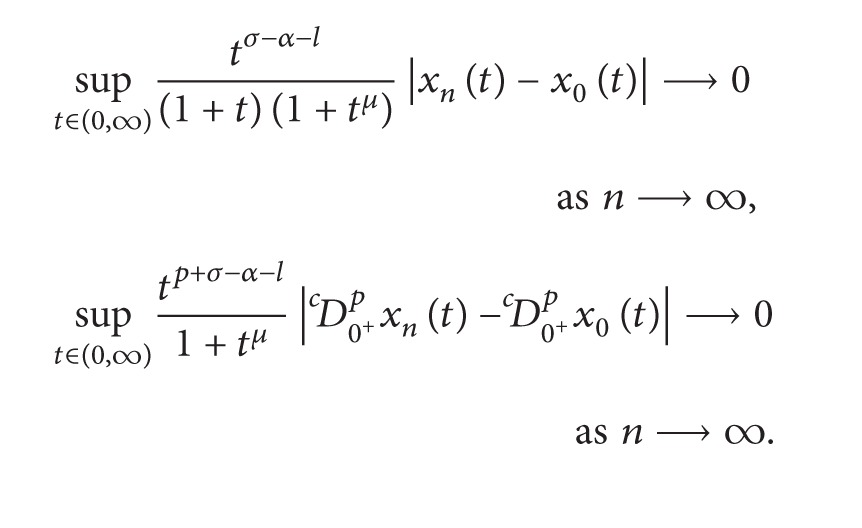

(54)

Since *f* : [0, *∞*) × *R* ^2^ → *R* is a Caratheodory function and {*I*_*k*_} is a Caratheodory function sequence, then there exist *M*_*r*_ > 0 and *M*_*rk*_ > 0 such that

(55) |f(t,xn(t),Dc0+pxn(t))|≤Mr, t∈[0,∞),|Ik(tk,xn(tk))|≤Mrk, k=1,2,3,…,∑k=1∞Mrk<∞.

Notice that

(56) (Txn)(t)=∫0t(t−s)α−1Γ(α)q(s)        ×f(s,xn(s),Dc0+pxn(s))ds       +x0+∑j=1kIj(tj,xn(tj)),       t∈(tk,tk+1], k=0,1,2,…,Dc0+p(Txn)(t)=∫0t(t−s)α−p−1Γ(α−p)q(s)           ×f(s,xn(s),Dc0+pxn(s))ds,              t∈(0,∞)∖{t1,t2,…}.

From the inequality

(57) $$\begin{array}{c} \sum_{j=1}^{\infty }|I_{j}\left(t_{j},x_{n}\left(t_{j}\right)\right)-I_{j}\left(t_{j},x_{0}\left(t_{j}\right)\right)|\leq 2\sum_{j=1}^{\infty }M_{rk}<\infty , \end{array}$$

it follows that there exists *N* > 0 for *ϵ* > 0 such that

(58) $$\begin{array}{c} \sum_{j=N}^{\infty }|I_{j}\left(t_{j},x_{n}\left(t_{j}\right)\right)-I_{j}\left(t_{j},x_{0}\left(t_{j}\right)\right)|<\epsilon . \end{array}$$

Since *x* → *I*_*k*_(*t*_*k*_, ((1 + *t*_*k*_)(1 + *t*_*k*_^−*α*−*l*^)/*t*_*k*_^−*α*−*l*^)*x*)  (*k* = 1,2,…, *N* − 1) is uniformly continuous on [−*r*, *r*], there exists *δ* > 0 such that

(59) |Ik(tk,(1+tk)(1+tkμ)tkσ−α−lx1) −Ik(tk,(1+tk)(1+tkμ)tkσ−α−lx2)|<ϵN−1

holds for all *x*_1_, *x*_2_ ∈ [−*r*, *r*] with |*x*_1_ − *x*_2_ | <*δ*, *k* = 1,2,…, *N* − 1. From (54), there exists *N*_1_ > *N* such that

(60) tσ−α−l(1+t)(1+tμ)|xn(t)−x0(t)|<δ,         t∈(0,∞),  n>N1,tp+σ−α−l1+tμ|Dc0+pxn(t)−Dc0+px0(t)|<δ,          t∈(0,∞), n>N1.

Hence,

(61) ∑j=1N−1|Ij(tj,xn(tj))−Ij(tj,x0(tj))| =∑j=1N−1|Ij(tj,(1+tj)(1+tjμ)tjσ−α−l‍        ×tjσ−α−l(1+tj)(1+tjμ)xn(tj))     −Ij(tj,(1+tj)(1+tjμ)tjσ−α−l         ×tjσ−α−l(1+tj)(1+tjμ)x0(tj))| <(N−1)ϵN−1=ϵ, n>N1.

Since

(62) tσ−α−l(1+t)(1+tμ)  ×∫0t(t−s)α−1Γ(α)|q(s)f(s,xn(s),Dc0+pxn(s))           −q(s)f(s,x0(s),Dc0+px0(s))|ds ≤2Mrtσ−α−l(1+t)(1+tμ)∫0t(t−s)α−1Γ(α)slds ≤2Mrtσ(1+t)(1+tμ)∫01(1−w)α−1Γ(α)wldw⟶0                      as  t⟶∞,

therefore, we can find *L* > 0 such that

(63) tσ−α−l(1+t)(1+tμ) ×∫0t(t−s)α−1Γ(α)   ×|q(s)f(s,xn(s),Dc0+pxn(s))     −q(s)f(s,x0(s),Dc0+px0(s))|ds<ϵ

holds for all *t* > *L*, *n* = 1,2,….

As *f* is a Caratheodory function, there exists *δ*_1_ > 0 such that

(64) |f(t,tσ−α−l(1+t)(1+tμ)u1,tp+σ−α−l1+tμv1) −f(t,tσ−α−l(1+t)(1+tμ)u2,tp+σ−α−l1+tμv2)|<ϵ

holds for all *t* ∈ [0, *L*] and *u*_1_, *u*_2_, *v*_1_, *v*_2_ ∈ [−*r*, *r*] with |*u*_1_ − *u*_2_ | <*δ*_1_, |*v*_1_ − *v*_2_ | <*δ*_1_. From (54), there exists *N*_2_ > *N* > *N*_1_ such that

(65) tσ−α−l(1+t)(1+tμ)|xn(t)−x0(t)|<δ1,        t∈(0,∞), n>N2,tp+σ−α−l1+tμ|Dc0+pxn(t)−Dc0+px0(t)|<δ1,         t∈(0,∞), n>N2.

So, for *t* ∈ [0, *L*], we have

(66) tσ−α−l(1+t)(1+tμ)  ×∫0t(t−s)α−1Γ(α)     ×|q(s)f(s,xn(s),Dc0+pxn(s))         −q(s)f(s,x0(s),Dc0+px0(s))|ds =tσ−α−l(1+t)(1+tμ)  ×∫0t(t−s)α−1Γ(α)    ×|q(s)f(s,(1+s)(1+sμ)sσ−α−l           ×sσ−α−l(1+s)(1+sμ)xn(s),           1+sμsp+σ−α−lsp+σ−α−l1+sμ           × cD0+pxn(s))       −q(s)f(s,(1+s)(1+sμ)sσ−α−l             ×sσ−α−l(1+s)(1+sμ)x0(s),             1+sμsp+σ−α−lsp+σ−α−l1+sμ             ×Dc0+px0(s))|ds ≤tσ−α−l(1+t)(1+tμ)∫0t(t−s)α−1Γ(α)slϵ ds =ϵtσ(1+t)(1+tμ)∫01(1−w)α−1Γ(α)wldw ≤Mσ,μB(α,l+1)Γ(α)ϵ, n>N2,  t∈[0,L].

Consequently, for all *n* > *N*_2_, *t* ∈ [0, *∞*), we get

(67) tσ−α−l(1+t)(1+tμ)  ×∫0t(t−s)α−1Γ(α)     ×|q(s)f(s,xn(s),Dc0+pxn(s))       −q(s)f(s,x0(s),Dc0+px0(s))|ds  <ϵ+Mσ,μB(α,l+1)Γ(α)ϵ.

In particular, for *t* ∈ (*t*_*k*_, *t*_*k*+1_], we find that

(68) tσ−α−l(1+t)(1+tμ)|(Txn)(t)−(Tx0)(t)| ≤tσ−α−l(1+t)(1+tμ)  ×∫0t(t−s)α−1Γ(α)    ×|f(s,xn(s),Dc0+pxn(s))        −f(s,x0(s),Dc0+px0(s))|ds  +∑j=1k|Ij(tj,xn(tj))−Ij(tj,x0(tj))| ≤ϵ+B(α,l+1)Γ(α)ϵ  +∑j=1N−1|Ij(tj,xn(tj))−Ij(tj,x0(tj))|  +∑j=N∞|Ij(tj,xn(tj))−Ij(tj,x0(tj))| <3ϵ+Mσ,μB(α,l+1)Γ(α)ϵ, n>N2.

Thus, it follows that

(69) sup⁡t∈(0,∞)tσ−α−l(1+t)(1+tμ)   ×|(Txn)(t)−(Tx0)(t)|⟶0 as  n⟶∞.

Similarly, it can be shown that

(70) sup⁡t∈(0,∞)tp+σ−α−l1+tμ|Dc0+(Txn)(t)−Dc0+(Tx0)(t)|⟶0                      as  n⟶∞.

From (69) and (70), we conclude that lim⁡_*n*→*∞*_ *Tx*_*n*_ = *Tx*_0_. This implies that *T* is continuous.

(b) Let us recall that *Ω* ⊂ *X* is relatively compact if it is bounded, both (*t* ^*σ*−*α*−*l*^/(1 + *t*)(1 + *t* ^*μ*^))*Ω* and (*t* ^*p*+*σ*−*α*−*l*^/(1 + *t* ^*μ*^)) ^*c*^ *D*_0^+^_^*p*^ *Ω* are equicontinuous on any closed subinterval [*a*, *b*] of (*t*_*k*_, *t*_*k*+1_]  (*k* = 0,1, 2,…) and equiconvergent at *t* = *t*_*k*_  (*k* = 0,1, 2,…), and *t* = *∞*.

Let *W* ⊂ *X* be a nonempty bounded set. To prove that *T* is completely continuous, we need to prove that *TW* is bounded, *TW* is equicontinuous on finite closed sub-interval on (*t*_*k*_, *t*_*k*+1_]  (*k* = 0,1, 2,…), *TW* is equiconvergent at *t* = *t*_*k*_  (*k* = 0,1, 2,…), and *TW* is equiconvergent at *t* = *∞*.

Since *W* is bounded, therefore, (49), (50), and (51) hold for *x* ∈ *W*. Following the method of proof for Lemma 8, it can easily be shown that *TW* is bounded.

Next we show that *TW* is equicontinuous on finite closed sub-interval on (*t*_*k*_, *t*_*k*+1_]  (*k* = 0,1, 2,…).

For [*a*, *b*]⊂(*t*_*k*_, *t*_*k*+1_] with *s*_1_, *s*_2_ ∈ [*a*, *b*] with *s*_1_ < *s*_2_ and *x* ∈ *W*, we have

(71) |s1σ−α−l(1+s1)(1+s1μ)  ×∫0s1(s1−s)α−1Γ(α)q(s)f(s,x(s),Dc0+px(s))ds  −s2σ−α−l(1+s2)(1+s2μ)  ×∫0s2(s2−s)α−1Γ(α)q(s)f(s,x(s),Dc0+px(s))ds| ≤|s1σ−α−l(1+s1)(1+s1μ)−s2σ−α−l(1+s2)(1+s2μ)|  ×∫0s2(s2−s)α−1Γ(α)|q(s)f(s,x(s),Dc0+px(s))|ds  +s1σ−α−l(1+s1)(1+s1μ)  ×∫s1s2(s2−s)α−1Γ(α)|q(s)f(s,x(s),Dc0+px(s))|ds  +s1σ−α−l(1+s1)(1+s1μ)  ×∫0s1|(s1−s)α−1−(s2−s)α−1|Γ(α)     ×|q(s)f(s,x(s),Dc0+px(s))|ds ≤|s1σ−α−l(1+s1)(1+s1μ)−s2σ−α−l(1+s2)(1+s2μ)|  ×Mr∫0s2(s2−s)α−1Γ(α)slds  +s1σ−α−l(1+s1)(1+s1μ)Mr∫s1s2(s2−s)α−1Γ(α)slds  +Mrs1σ−α−l(1+s1)(1+s1μ)  ×∫0s1|(s1−s)α−1−(s2−s)α−1|Γ(α)slds =|s1σ−α−l(1+s1)(1+s1μ)−s2σ−α−l(1+s2)(1+s2μ)|  ×s2α+lMr∫01(1−w)α−1Γ(α)wldw  +s1σ−α−l(1+s1)(1+s1μ)Mrs2α+l  ×∫s1/s21(1−w)α−1Γ(α)wldw  +Mrs1σ−α−l(1+s1)(1+s1μ)  ×∫0s1(s1−s)α−1−(s2−s)α−1Γ(α)slds ≤|s1σ−α−l(1+s1)(1+s1μ)−s2σ−α−l(1+s2)(1+s2μ)|  ×s2α+lMr∫01(1−w)α−1Γ(α)wldw  +Mrmax⁡{aα+l,bα+l}∫s1/s21(1−w)α−1Γ(α)wldw  +Mr[s1α+l∫01(1−w)α−1Γ(α)wldw       −s2α+l∫0s1/s2(1−w)α−1Γ(α)wldw] ≤|s1σ−α−l(1+s1)(1+s1μ)−s2σ−α−l(1+s2)(1+s2μ)|  ×max⁡{aα+l,bα+l}Mr∫01(1−w)α−1Γ(α)wldw  +Mrmax⁡{aα+l,bα+l}∫s1/s21(1−w)α−1Γ(α)wldw  +Mr|s1α+l−s2α+l|∫01(1−w)α−1Γ(α)wldw  +max⁡⁡{aα+l,bα+l}∫s1/s21(1−w)α−1Γ(α)wldw ⟶0     uniformly  as  s1⟶s2  withs1,s2∈[a,b]⊂(tk,tk+1].

So

(72) |s1σ−α−l(1+s1)(1+s1μ)(Tx)(s1)−s2σ−α−l(1+s2)(1+s2μ)(Tx)(s2)| =|s1σ−α−l(1+s1)(1+s1μ)∫0s1(s1−s)α−1Γ(α)q(s)               ×f(s,x(s),Dc0+px(s))ds   −s2σ−α−l(1+s2)(1+s2μ)∫0s2(s2−s)α−1Γ(α)q(s)               ×f(s,x(s),Dc0+px(s))ds|  +|x0||s1σ−α−l(1+s1)(1+s1μ)−s2σ−α−l(1+s2)(1+s2μ)|  +|s1σ−α−l(1+s1)(1+s1μ)−s2σ−α−l(1+s2)(1+s2μ)|  ×∑j=1k|Ij(tj,x(tj))| ≤|s1σ−α−l(1+s1)(1+s1μ)∫0s1(s1−s)α−1Γ(α)q(s)               ×f(s,x(s),Dc0+px(s))ds   −s2σ−α−l(1+s2)(1+s2μ)∫0s2(s2−s)α−1Γ(α)q(s)              ×f(s,x(s),Dc0+px(s))ds|  +|x0||s1σ−α−l(1+s1)(1+s1μ)−s2σ−α−l(1+s2)(1+s2μ)|  +|s1σ−α−l(1+s1)(1+s1μ)−s2σ−α−l(1+s2)(1+s2μ)|∑j=1∞Mrk ⟶0  uniformly  as  s1⟶s2  with  s1,s2∈[a,b]⊂(tk,tk+1].

Thus,

(73) |s1σ−α−l(1+s1)(1+s1μ)(Tx)(s1) −s2σ−α−l(1+s2)(1+s2μ)(Tx)(s2)|⟶0

uniformly as *s*_1_ → *s*_2_ with *s*_1_, *s*_2_ ∈ [*a*, *b*]⊂(*t*_*k*_, *t*_*k*+1_]. Similarly, we have

(74) |s1p+σ−α−l1+s1μ cD0+p(Tx)(s1)−s2p+σ−α−l1+s2μ cD0+p(Tx)(s2)| =|s1p+σ−α−l1+s1μ∫0s1(s1−s)α−p−1Γ(α−p)q(s)         ×f(s,x(s),Dc0+px(s))ds   −s2p+σ−α−l1+s2μ∫0s2(s2−s)α−p−1Γ(α−p)q(s)          ×f(s,x(s),Dc0+px(s))ds| ⟶0   uniformly  as  s1⟶s2  with  s1,s2∈[a,b]⊂(tk,tk+1].

From (73) and (74), we conclude that *TW* is equicontinuous on finite closed interval on (*t*_*k*_, *t*_*k*+1_].

Now wee prove that *TW* is equiconvergent as *t* → *t*_*k*_^+^  (*k* = 0,1, 2,…). For *μ* > *σ* > 0, we find that

(75) tσ−α−l(1+t)(1+tμ)|(Tx)(t)−x0| ≤tσ−α−l(1+t)(1+tμ)  ×∫0t(t−s)α−1Γ(α)q(s)     ×f(s,x(s),Dc0+px(s))ds ≤Mrtσ(1+t)(1+tμ)∫01(1−w)α−1Γ(α)wldw ⟶0 uniformly  in  W  as  t⟶0,tp+σ−α−l1+tμ|Dc0+p(Tx)(t)| ≤tp+σ−α−l1+tμ  ×∫0t(t−s)α−p−1Γ(α−p)     ×|q(s)f(s,x(s),Dc0+px(s))|ds ≤Mrtp+σ−α−l1+tμ∫0t(t−s)α−p−1Γ(α−p)slds =Mrtσ1+tμ∫01(1−w)α−p−1Γ(α−p)wldw ⟶0 uniformly  in  W  as  t⟶0.

It follows that

(76) tp+σ−α−l1+tμ|Dc0+p(Tx)(t)|⟶0 uniformly  in  W  as  t⟶0.

From (75), it follows that *TW* is equiconvergent as *t* → 0^+^.

For *t* → *t*_*k*_^+^  (*t* ∈ (*t*_*k*_, *t*_*k*+1_], *k* = 1,2,…), we have

(77) tσ−α−l(1+t)(1+tμ) ×|(Tx)(t)(∫0tk(tk−s)α−1Γ(α)q(s)          ×f(s,x(s),Dc0+px(s))ds         +x0+∑j=1kIj(tj,x(tj)))| ≤tσ−α−l(1+t)(1+tμ)  ×|∫0t(t−s)α−1Γ(α)q(s)f(s,x(s),Dc0+px(s))ds    −∫0tk(tk−s)α−1Γ(α)q(s)f(s,x(s),Dc0+px(s))ds| ≤tσ−α−l(1+t)(1+tμ)  ×∫tkt(tk−s)α−1Γ(α)|q(s)f(s,x(s),Dc0+px(s))|ds  +tσ−α−l(1+t)(1+tμ)  ×∫0tk|(tk−s)α−1−(t−s)α−1|Γ(α)     ×|q(s)f(s,x(s), cD0+px(s))|ds ≤Mrtσ(1+t)(1+tμ)  ×∫tk/t1(1−w)α−1Γ(α)wldw  +Mrtσ−α−l(1+t)(1+tμ)  ×[tkα+l∫01(1−w)α−1Γ(α)wldw    −∫0tk/t(1−w)α−1Γ(α)wldw] ⟶0 uniformly  in  W  as  t⟶tk+,tp+σ−α−l1+tμ|Dc0+(Tx)(t)        −∫0tk(tk−s)α−p−1Γ(α−p)q(s)         ×f(s,x(s),Dc0+px(s))ds| ≤tp+σ−α−l1+tμ∫tkt(t−s)α−p−1Γ(α−p)         ×|q(s)f(s,x(s),Dc0+px(s))|ds  +tp+σ−α−l1+tμ  ×∫0tk|(tk−s)α−p−1−(t−s)α−p−1|Γ(α−p)     ×|q(s)f(s,x(s),Dc0+px(s))|ds ≤Mrtp+σ−α−l1+tμ∫tkt(t−s)α−p−1Γ(α−p)slds  +Mrtp+σ−α−l1+tμ  ×∫0tk|(tk−s)α−p−1−(t−s)α−p−1|Γ(α−p)slds ≤MrMσ,μ∫tk/t1(1−w)α−p−1Γ(α−p)wldw  +Mrtp+σ−α−l1+tμ  ×[tkα−p+l∫01(1−w)α−p−1Γ(α−p)wldw     −tα−p+l∫0tk/t(1−w)α−p−1Γ(α−p)wldw] ≤MrMσ,μ∫tk/t1(1−w)α−p−1Γ(α−p)wldw  +Mrtp+σ−α−l1+tμ  ×[(tkα−p+l−tα−p+l)B(α−p,l+1)Γ(α−p)    +tα−p+l∫tk/t1(1−w)α−p−1Γ(α−p)wldw] ≤MrMσ,μ∫tk/t1(1−w)α−p−1Γ(α−p)wldw  +MrMp+σ−α−l,μ  ×[(tkα−p+l−tα−p+l)B(α−p,l+1)Γ(α−p)    +max⁡{tkα−p+l,tk+1α−p+l}∫tk/t1(1−w)α−p−1Γ(α−p)wldw] ⟶0 uniformly  in  W  as  t⟶tk+,

which imply that *TW* is equiconvergent as *t* → *t*_*k*_^+^  (*k* = 1,2, 3,…).

Our next task is to show that *TW* is equiconvergent as *t* → *∞*. Observe that

(78) tσ−α−l(1+t)(1+tμ)  ×|(Tx)(t)−(x0+∑j=1∞Ij(tj,x(tj)))| ≤tσ−α−l(1+t)(1+tμ)  ×∫0t(t−s)α−1Γ(α)q(s)f(s,x(s),Dc0+px(s))ds ≤Mrtσ(1+t)(1+tμ)∫01(1−w)α−1Γ(α)wldw ⟶0 uniformly  in  Ω1  as  t⟶∞,tp+σ−α−l1+tμ|Dc0+p(Tx)(t)| ≤tp+σ−α−l1+tμ∫0t(t−s)α−p−1Γ(α)Mrslds ⟶0 uniformly  in  W  as  t⟶∞.

Hence, *TW* is equiconvergent as *t* → *∞*.

From the above steps, it follows that *T* is completely continuous. This completes the proof.

In the sequel, we need the following assumption:

(*H* _1_) *f* is a Caratheodory function such that
(79) |f(t,(1+t)(1+tμ)tσ−α−lu1,1+tμtp+σ−α−lu2)−C|  ≤∑i=1mAi|u1|δi+∑i=1mBi|u2|δi,
 where 0 < *δ*_1_ < *δ*_2_ < ⋯<*δ*_*m*_ and *A*_*i*_, *B*_*i*_  (*i* = 1,2,…, *m*), *C* ≥ 0 are real numbers;
(*H* _2_) {*I*_*k*_}  (*k* = 1,2,…) is a Caratheodory sequence and there exist numbers *A*_*ki*_ ≥ 0  (*i* = 1,2,…, *m*), *D*_*k*_ ≥ 0  (*k* = 1,2,…), *δ*_*i*_ ≥ 0  (*i* = 1,2,…, *m*) such that
(80) |Ik(tk,(1+tk)(1+tkμ)tkσ−α−lu)−Dk|  ≤∑i=1mAki|u|δi,k=1,2,3,…  holds  ∀t∈(0,∞),  u∈R.

Furthermore, we set

(81) $$\begin{array}{c} M_{0}=\max\left\{M_{1},M_{2}\right\}, \end{array}$$

where

(82) M1=∑i=1m[Mσ,μB(α,l+1)Γ(α)[Ai+Bi]    +Mσ−α−l,μ∑j=1∞Aji]||Ψ||δi−δm,

(83) $$\begin{array}{c} M_{2}=M_{\sigma ,\mu }\frac{B\left(\alpha -p,l+1\right)}{\Gamma \left(\alpha -p\right)}\sum_{i=1}^{m}\left[A_{i}+B_{i}\right]. \end{array}$$

**Theorem 11**

Suppose that (*H*_1_) and (*H*_2_) hold. Then IVP (6) has at least one solution *x* ∈ *X* if

(84) δm<1 or  δm=1 with  M0<1 or  δm>1 with  ||Ψ||1−δm(δm−1)δm−1δmδm≥M0.

**Proof**

Let *X* be the Banach space as defined in Section 2 and let *T* : *X* → *X* be an operator given by (98). In view of Lemma 8, it follows from the assumptions (*H*_1_) and (*H*_2_) that *T* is well defined and is completely continuous. Thus, we seek solutions of IVP (6) by finding fixed points of *T* in *X*.

Let us introduce

(85) Ψ(t)=C∫0t(t−s)α−1Γ(α)q(s)ds+x0+∑j=1kDj, t∈(tk,tk+1],  k=0,1,2,….

It is easy to show that Ψ ∈ *X*. For *r* > 0, we define ${\bar{\Omega }}_{r}=\left\{x\in X:||x-\Psi ||\leq r\right\}$ . Then, for $x\in {\bar{\Omega }}_{r}$ , we have

(86) ||x||=max⁡{sup⁡t∈(0,∞)tσ−α−l(1+t)(1+tμ)|x(t)|,    sup⁡t∈(0,∞)tp+σ−α−l1+tμ| cD0+px(t)|}≤||x−Ψ||+||Ψ||≤r+||Ψ||.

Using the assumptions (*H*_1_) and (*H*_2_), we find that

(87) tσ−α−l(1+t)(1+tμ)|(Tx)(t)−Ψ(t)| ≤tσ−α−l(1+t)(1+tμ)  ×∫0t(t−s)α−1Γ(α)|q(s)|     ×|f(s,x(s),Dc0+px(s))−C|ds  +tσ−α−l(1+t)(1+tμ)∑j=1k|Ij(tj,x(tj))−Dj| ≤tσ−α−l(1+t)(1+tμ)  ×∫0t(t−s)α−1Γ(α)sl     ×[∑i=1mAi|tσ−α−l(1+t)(1+tμ)x(s)|δi       +∑i=1mBi|tp+σ−α−l1+tμ cD0+px(s)|δi]ds  +tσ−α−l(1+t)(1+tμ)  ×∑j=1k ∑i=1mAji|tjσ−α−l(1+tj)(1+tjμ)x(tj)|δi ≤tσ(1+t)(1+tμ)  ×∫01(1−w)α−1Γ(α)wl    ×[∑i=1mAi||x||δi+∑i=1mBi||x||δi]dw  +Mσ−α−l∑j=1k ∑i=1mAji||x||δi ≤Mσ,μB(α,l+1)Γ(α)∑i=1m[Ai+Bi]||x||δi  +Mσ−α−l∑j=1k ∑i=1mAji||x||δi =∑i=1m[Mσ,μB(α,l+1)Γ(α)[Ai+Bi]      +Mσ−α−l∑j=1kAji]||x||δi ≤[r+||Ψ||]δm∑i=1m[Mσ,μB(α,l+1)Γ(α)[Ai+Bi]            +Mσ−α−l∑j=1kAji]  ×[r+||Ψ||]δi−δm ≤[r+||Ψ||]δm  ×∑i=1m[Mσ,μB(α,l+1)Γ(α)[Ai+Bi]         +Mσ−α−l,μ∑j=1∞Aji]||Ψ||δi−δm ≤M1[r+||Ψ||]δm,tp+σ−α−l1+tμ| cD0+p(Tx)(t)−Dc0+pΨ(t)| ≤tp+σ−α−l1+tμ∫0t(t−s)α−p−1Γ(α−p)|q(s)|        ×|f(s,x(s),Dc0+px(s))−C|ds ≤tp+σ−α−l1+tμ  ×∫0t(t−s)α−p−1Γ(α−p)sl    ×[∑i=1mAi|tσ−α−l(1+t)(1+tμ)x(s)|δi      +∑i=1mBi|tp+σ−α−l1+tμDc0+px(s)|δi]ds ≤tp+σ−α−l1+tμ  ×∫0t(t−s)α−p−1Γ(α−p)sl    ×[∑i=1mAi||x||δi+∑i=1mBi||x||δi]ds ≤Mσ,μB(α−p,l+1)Γ(α−p)  ×[∑i=1mAi||x||δi+∑i=1mBi||x||δi] ≤Mσ,μB(α−p,l+1)Γ(α−p)∑i=1m[Ai+Bi]||x||δi ≤Mσ,μB(α−p,l+1)Γ(α−p)  ×∑i=1m[Ai+Bi][r+||Ψ||]δi ≤[r+||Ψ||]δmM2.

Thus, by (81), it follows that

(88) $$\begin{array}{c} ||Tx-\Psi ||\leq {\left[r+||\Psi ||\right]}^{\delta_{m}}M_{0}. \end{array}$$

Next, we have the following cases.

(i) For *δ*_*m*_ < 1, we can choose *r*_0_ > 0 sufficiently large such that [*r*_0_ + ||Ψ||]^*δ*_*m*_^ *M*_0_ < *r*_0_. Let *Ω*_*r*_0__ = {*x* ∈ *X* : ||*x*|| < *r*_0_}. It is easy to see that $T{\bar{\Omega }}_{r_{0}}\subset {\bar{\Omega }}_{r_{0}}$ . Then, by Schauder's fixed point theorem, the operator *T* has a fixed point $x\in {\bar{\Omega }}_{r_{0}}$ , which is a bounded solution of IVP (6).

(ii) In case *δ*_*m*_ = 1, we choose

(89) $$\begin{array}{c} r_{0}\geq \frac{||\Psi ||M_{0}}{1-M_{0}}. \end{array}$$

Let *Ω*_*r*_0__ = {*x* ∈ *X* : ||*x*|| < *r*_0_}. Then it can easily be shown that $T{\bar{\Omega }}_{r_{0}}\subset {\bar{\Omega }}_{r_{0}}$ . Thus, Schauder's fixed point theorem applies and the operator *T* has a fixed point $x\in {\bar{\Omega }}_{r_{0}}$ , which is a bounded solution of IVP (6).

(iii) For *δ*_*m*_ > 1, we choose *r* = *r*_0_ = ||Ψ||/(*δ*_*m*_ − 1) such that

(90) $$\begin{array}{c} \frac{r_{0}}{{\left(r_{0}+||\Psi ||\right)}^{\delta_{m}}}=\frac{{||\Psi ||}^{1-\delta_{m}}{\left(\delta_{m}-1\right)}^{\delta_{m}-1}}{\delta_{m}^{\delta_{m}}}\geq M_{0}. \end{array}$$

Let *Ω*_*r*_0__ = {*x* ∈ *X* : ||*x*|| < *r*_0_}. As before, it is easy to show that $T{\bar{\Omega }}_{r_{0}}\subset {\bar{\Omega }}_{r_{0}}$ . Then, it follows from Schauder's fixed point theorem that *T* has a fixed point $x\in {\bar{\Omega }}_{r_{0}}$ , which corresponds to a solution of IVP (6). This completes the proof.

**Theorem 12**

Suppose that (*H*_1_) and (*H*_2_) hold with *δ*_*m*_ = 1. Then IVP (6) has a unique solution *x* ∈ *X* if *M*_0_ < 1.

**Proof**

By Theorem 11, IVP (6) has at least one solution. Let *x*_1_ and *x*_2_ be two different solutions of IVP (6). Then ||*x*_1_ − *x*_2_|| > 0,  *Tx*_1_ = *x*_1_, and *Tx*_2_ = *x*_2_. Employing the method used in the proof of Theorem 11, we find that

(91) $$\begin{array}{c} \frac{t^{\sigma -\alpha -l}}{\left(1+t\right)\left(1+t^{\mu }\right)}\left|\left(Tx_{1}\right)\left(t\right)-\left(Tx_{2}\right)\left(t\right)\right|\leq M_{1}||x_{1}-x_{2}||,\\ \frac{t^{p+\sigma -\alpha -l}}{1+t^{\mu }}\left|{\;}^{c}D_{0^{+}}^{p}\left(Tx_{1}\right)\left(t\right)-{\;}^{c}D_{0^{+}}^{p}\left(Tx_{2}\right)\left(t\right)\right|\leq M_{2}||x_{1}-x_{2}||. \end{array}$$

Thus, ||*Tx*_1_ − *Tx*_2_|| ≤ *M*_0_||*x*_1_ − *x*_2_||. On the other hand, by (51), we get

(92) $$\begin{array}{c} 0<||x_{1}-x_{2}||=||Tx_{1}-Tx_{2}||\leq M_{0}||x_{1}-x_{2}||<||x_{1}-x_{2}||, \end{array}$$

which is a contradiction. Hence, IVP (6) has a unique solution *x* ∈ *X* if *M*_0_ < 1. This completes the proof.

Next, consider the following IVP:

(93) Dc0+αx(t)=q(t)f(t,x(t),Dc0+px(t)), t∈(0,∞),x(0)=x0,Δx(tk)=Ik(tk,x(tk)), k=1,2,…,

where *x*_0_, *a*_*k*_  (*k* = 1,2,…) are constants, ∑_*k*=1_^*∞*^|*a*_*k*_| is convergent, and *f* is a Caratheodory function; there exists *l* ∈ (−1, −*α*) such that |*q*(*t*)|≤*t* ^*l*^ for all *t* ∈ (0, *∞*).

**Theorem 13**

Assume that the conditions (*H*_1_) and (*H*_2_) hold. Then every solution of (93) tends to *x*_0_ + ∑_*k*=1_^*∞*^ *a*_*k*_ as *t* → *∞* provided that (84) is satisfied.

**Proof**

By Theorem 11, there exist solutions for IVP (93) satisfying the integral equation

(94) x(t)=∫0t(t−s)α−1Γ(α)q(s)f(s,x(s), cD0+px(s))ds+x0+∑j=1kaj, t∈(tk,tk+1],  k=0,1,2,….

Clearly,

(95) max⁡{sup⁡t∈(0,∞)tσ−α−l(1+t)(1+tμ)|x(t)|,    sup⁡t∈(0,∞)tp+σ−α−l1+tμ| cD0+px(t)|}≤r<∞.

Since *f* is a Caratheodory function by (*H*_1_), therefore, there exists *M*_*r*_ > 0 such that

(96) $$\begin{array}{c} \left|f\left(t,x\left(t\right),{\;}^{c}D_{0^{+}}^{p}x\left(t\right)\right)\right|\leq M_{r},\;t\in \left[0,\infty \right). \end{array}$$

So

(97) |x(t)−(x0+∑j=1∞aj)| =|∫0t(t−s)α−1Γ(α)q(s)       ×f(s,x(s),Dc0+px(s))ds+∑j=k+1∞aj| ≤Mr∫0t(t−s)α−1Γ(α)slds+∑j=k+1∞|aj| =Mrtα+l∫01(1−w)α−1Γ(α)wldw   +∑j=k+1∞|aj|⟶0 as  t⟶∞.

This completes the proof.

## 4. Existence of Solutions for an IVP with Multiple Base Points

In this section, we show the existence for solutions for IVP (7) with multiple base points. Let us introduce an operator *T*_*m*_ on *Y* as

(98) (Tmx)(t)=∫tkt(t−s)α−1Γ(α)q(s)f(s,x(s),Dctk+px(s))ds+x0+∑j=1kIj(tj,x(tj))+∑j=1k∫tj−1tj(tj−s)α−1Γ(α)q(s)    ×f(s,x(s),Dctj−1+px(s))ds,    t∈(tk,tk+1], k=0,1,2,….

**Lemma 14**

Suppose that *f* is a Caratheodory function and {*I*_*k*_} is a Caratheodory function sequence and *λ*_0_ = :inf⁡_*k*=1,2,3,…_⁡(*t*_*k*_ − *t*_*k*−1_) > 0. Then

- *T*_*m*_ : *Y* → *Y* is well defined;
- the fixed point of the operator *T*_*m*_ coincides with the solution of IVP (7);
- *T*_*m*_ : *Y* → *Y* is completely continuous.

**Proof**

(i) For *x* ∈ *Y*, we set

(99) r=max⁡{sup⁡t∈(0,∞)tσ−α−l(1+t)(1+tμ)|x(t)|,    sup⁡k=0,1,2,… sup⁡t∈(tk,tk+1]tp+σ−α−l1+tμ|Dctk+px(t)|}<+∞.

Since *f* is a Caratheodory function, {*I*_*k*_} is Caratheodory function sequence; there exist positive numbers ${\tilde{M}}_{r}>0$ and *M*_*rk*_ > 0  (*k* = 1,2,…) such that

(100) |f(t,x(t),Dctk+px(t))|≤M~r, t∈[0,∞), |Ik(tk,x(tk))|≤Mrk, k=1,2,…,  ∑k=1∞Mrk<∞.

It is easy to show that

(101) Tmx|(tk,tk+1]∈C0(tk,tk+1],Dctk+pTmx|(tk,tk+1]∈C0(tk,tk+1],          k=0,1,2,….

As in Lemma 9, we can show that

(102) $$\begin{array}{c} \frac{t^{\sigma -\alpha -l}}{\left(1+t\right)\left(1+t^{\mu }\right)}\left(T_{m}x\right)\left(t\right)\;\;\text{is}\;\text{bounded},\\ {\left\{\underset{t\in (t_{k},t_{k+1}]}{\sup}\frac{t^{p+\sigma -\alpha -l}}{1+t^{\mu }}{\;}^{c}D_{t_{k}^{+}}^{p}(T_{m}x)(t)\right\}}_{k=0}^{\infty }\;\text{is}\;\text{bounded}. \end{array}$$

Hence, *T*_*m*_ *x* ∈ *Y*. This implies that *T*_*m*_ : *Y* → *Y* is well defined.

(ii) It follows from Lemma 9 that the fixed point of the operator *T*_*m*_ coincides with the solution of IVP (7).

(iii) To show that *T*_*m*_ is completely continuous, we split the proof into several steps.

*Step 1.* *T*_*m*_ is continuous.

Let *x*_*n*_ ∈ *Y* with *x*_*n*_ → *x*_0_ as *n* → *∞*. We will prove that *T*_*m*_ *x*_*n*_ → *T*_*m*_ *x*_0_ as *n* → *∞*. It is easy to see that there exists *r* > 0 such that


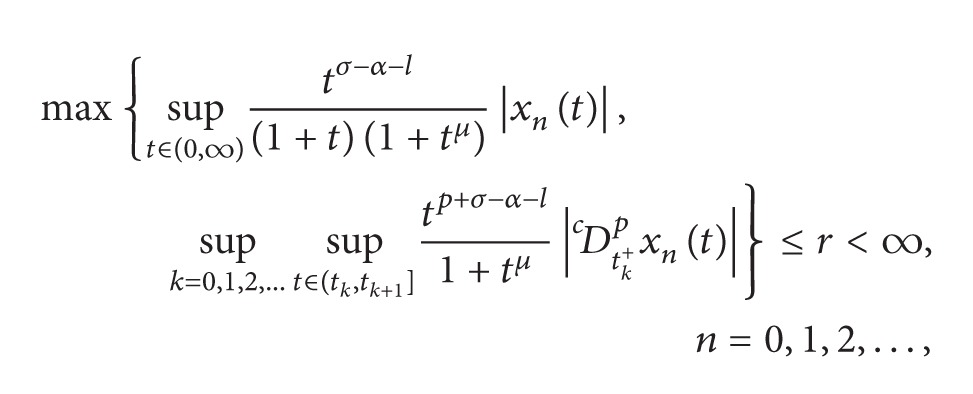

(103)



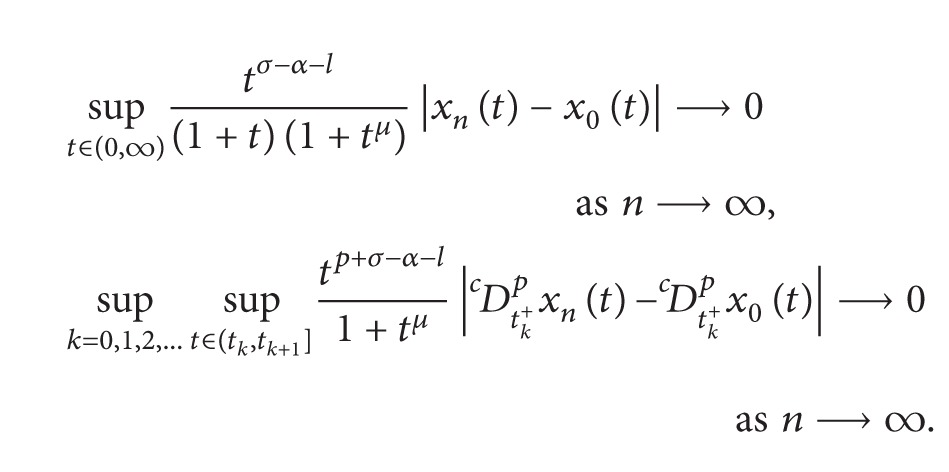

(104)

As in the proof of Lemma 10,

(105) $$\begin{array}{c} \sum_{j=1}^{N-1}\left|I_{j}\left(t_{j},x_{n}\left(t_{j}\right)\right)-I_{j}\left(t_{j},x_{0}\left(t_{j}\right)\right)\right|<\epsilon ,\;n>N_{1}. \end{array}$$

From *λ*_0_ = inf⁡_*k*=1,2,…_⁡(*t*_*k*_ − *t*_*k*−1_) > 0, we get *t*_*k*_ > *kλ*_0_ for all *k* = 0,1, 2,….

Since ∑_*j*=*K*+1_^*∞*^(1/*j* ^*μ*+1−*σ*^) is convergent, there exists *K* > 0 such that

(106) $$\begin{array}{c} \sum_{j=K+1}^{\infty }\frac{1}{j^{\mu +1-\sigma }}<\epsilon . \end{array}$$

Then

(107) tσ−α−l(1+t)(1+tμ) ×∫tkt(t−s)α−1Γ(α)    ×|q(s)f(s,xn(s),Dctk+pxn(s))       −q(s)f(s,x0(s),Dctk+px0(s))|ds +tσ−α−l(1+t)(1+tμ) ×∑j=K+1k∫tj−1tj(tj−s)α−1Γ(α)      ×|q(s)f(s,xn(s),Dctk+pxn(s))        −q(s)f(s,x0(s),Dctk+px0(s))|ds  ≤2M~rtσ−α−l(1+t)(1+tμ)∫tkt(t−s)α−1Γ(α)slds  +2Mrtσ−α−l(1+t)(1+tμ)  ×∑j=K+1k∫tj−1tj(tj−s)α−1Γ(α)slds  ≤2M~r1tμ+1−σ∫tk/t1(1−w)α−1Γ(α)wldw  +2M~r1tμ+1−σ+α+l  ×∑j=K+1ktjα+l∫tj−1/tj1(1−w)α−1Γ(α)wldw  ≤2M~r1tkμ+1−σ∫01(1−w)α−1Γ(α)wldw  +2M~r∑j=K+1k1tjμ+1−σ+α+ltjα+l  ×∫01(1−w)α−1Γ(α)wldw  ≤4M~r∑j=K+1k1tjμ+1−σB(α,l+1)Γ(α)  =4MrB(α,l+1)Γ(α)∑j=K+1k1tjμ+1−σ  ≤4M~rB(α,l+1)Γ(α)∑j=K+1k1(jλ0)μ+1−σ  =4M~rB(α,l+1)Γ(α)1λ0μ+1−σ∑j=K+1∞1jμ+1−σ  ≤4M~rB(α,l+1)Γ(α)1λ0μ+1−σϵ.

Since *f* is a Caratheodory function, there exists *δ*_1_ > 0 such that

(108) |f(t,tσ−α−l(1+t)(1+tμ)u1,tp+σ−α−l1+tμv1)  −f(t,tσ−α−l(1+t)(1+tμ)u2,tp+σ−α−l1+tμv2)| <ϵ∑j=1K(1/tjμ+1−σ)

holds for all *t* ∈ [0, *t*_*K*+1_] and *u*_1_, *u*_2_ ∈ [−*r*, *r*] with |*u*_1_ − *u*_2_ | <*δ*_1_, |*v*_1_ − *v*_2_ | <*δ*_1_. From (104), there exists *N*_2_ > *N*_1_ such that

(109) tσ−α−l(1+t)(1+tμ)|xn(t)−x0(t)|<δ1,         t∈(0,∞),  n>N2,tp+σ−α−l1+tμ|Dctk+pxn(t)−Dctk+px0(t)|<δ1,          t∈(tk,tk+1], n>N2.

So, for *t* ∈ [*t*_*k*_, *t*_*k*+1_], we have

(110) tσ−α−l(1+t)(1+tμ) ×∑j=1K∫tj−1tj(tj−s)α−1Γ(α)      ×|q(s)f(s,xn(s),Dctk+pxn(s))        −q(s)f(s,x0(s),Dctk+px0(s))|ds ≤tσ−α−l(1+t)(1+tμ)  ×∑j=1K∫tj−1tj(tj−s)α−1Γ(α)       ×slϵ∑j=1K(1/tjμ+1−σ)ds ≤ϵ∑j=1K(1/tjμ+1−σ)1tμ+1−σ+α+l  ×∑j=1Ktjα+l∫tj−1/tj1(1−w)α−1Γ(α)wldw ≤ϵ∑j=1K(1/tjμ+1−σ)  ×∑j=1K1tjμ+1−σ∫01(1−w)α−1Γ(α)wldw ≤B(α,l+1)Γ(α)ϵ, n>N2.

Thus, for *t* ∈ (*t*_*k*_, *t*_*k*+1_]  (*k* = 0,1, 2,…) with *n* > *N*_2_, we have

(111) tσ−α−l(1+t)(1+tμ)|(Tmxn)(t)−(Tmx0)(t)| ≤tσ−α−l(1+t)(1+tμ)  ×∫tkt(t−s)α−1Γ(α)  ×|f(s,xn(s),Dctk+pxn(s))    −f(s,x0(s),Dctk+px0(s))|ds  +∑j=1∞|Ij(tj,xn(tj))−Ij(tj,x0(tj))|  +tσ−α−l(1+t)(1+tμ)  ×∑j=1k∫tj−1tj(tj−s)α−1Γ(α)|q(s)|  ×|f(s,xn(s),Dctj−1+pxn(s))    −f(s,x0(s),Dctj−1+px0(s))|ds ≤3ϵ+4MrB(α,l+1)Γ(α)1λ0μ+1−σϵ  +B(α,l+1)Γ(α)ϵ, n>N2.

In consequence,

(112) sup⁡k=0,1,2,… sup⁡t∈(tk,tk+1]tσ−α−l(1+t)(1+tμ)  ×|(Tmxn)(t)−(Tmx0)(t)|⟶0 as  n⟶∞.

Similarly, we can show that

(113) sup⁡k=0,1,2,… sup⁡t∈(tk,tk+1]tp+σ−α−l1+tμ  ×|Dctk+(Tmxn)(t)−Dctk+(Tmx0)(t)|⟶0                   as  n⟶∞.

From (112) and (113), it follows that lim⁡_*n*→*∞*_ *T*_*m*_ *x*_*n*_ = *T*_*m*_ *x*_0_ which implies that *T*_*m*_ is continuous.

Let *W* ⊂ *X* be a nonempty bounded set. To prove that *T*_*m*_ is completely continuous, we need to prove that *T*_*m*_ *W* is bounded, *T*_*m*_ *W* is equicontinuous on finite closed sub-interval on (*t*_*k*_, *t*_*k*+1_]  (*k* = 0,1, 2…), *T*_*m*_ *W* is equiconvergent at *t* = *t*_*k*_  (*k* = 0,1, 2,…), and *T*_*m*_ *W* is equiconvergent at *t* = *∞*.

*Step 2.* As in the proof of Lemma 10, it is easy to show that *T*_*m*_ *W* is bounded.

*Step 3.* We prove that *T*_*m*_ *W* is equicontinuous on finite closed sub-interval on (*t*_*k*_, *t*_*k*+1_]  (*k* = 0,1, 2,…). For [*a*, *b*]⊂(*t*_*k*_, *t*_*k*+1_] with *s*_1_, *s*_2_ ∈ [*a*, *b*] with *s*_1_ < *s*_2_ and *x* ∈ *W*, we have

(114) |s1σ−α−l(1+s1)(1+s1μ) ×∫tks1(s1−s)α−1Γ(α)q(s)    ×f(s,x(s),Dctk+px(s))ds −s2σ−α−l(1+s2)(1+s2μ) ×∫tks2(s2−s)α−1Γ(α)q(s)    ×f(s,x(s),Dctk+px(s))ds| ≤|s1σ−α−l(1+s1)(1+s1μ)−s2σ−α−l(1+s2)(1+s2μ)|  ×∫tks2(s2−s)α−1Γ(α)     ×|q(s)f(s,x(s),Dctk+px(s))|ds  +s1σ−α−l(1+s1)(1+s1μ)  ×∫s1s2(s2−s)α−1Γ(α)     ×|q(s)f(s,x(s),Dctk+px(s))|ds  +s1σ−α−l(1+s1)(1+s1μ)  ×∫tks1|(s1−s)α−1−(s2−s)α−1|Γ(α)    ×|q(s)f(s,x(s),Dctk+px(s))|ds ≤|s1σ−α−l(1+s1)(1+s1μ)−s2σ−α−l(1+s2)(1+s2μ)|  ×M~r∫tks2(s2−s)α−1Γ(α)slds  +s1σ−α−l(1+s1)(1+s1μ)M~r∫s1s2(s2−s)α−1Γ(α)slds  +M~rs1σ−α−l(1+s1)(1+s1μ)  ×∫tks1|(s1−s)α−1−(s2−s)α−1|Γ(α)slds =|s1σ−α−l(1+s1)(1+s1μ)−s2σ−α−l(1+s2)(1+s2μ)|s2α+l  ×M~r∫01(1−w)α−1Γ(α)wldw  +s1σ−α−l(1+s1)(1+s1μ)M~rs2α+l∫s1/s21(1−w)α−1Γ(α)wldw  +M~rs1σ−α−l(1+s1)(1+s1μ)  ×∫tks1(s1−s)α−1−(s2−s)α−1Γ(α)slds ≤|s1σ−α−l(1+s1)(1+s1μ)−s2σ−α−l(1+s2)(1+s2μ)|  ×s2α+lM~r∫01(1−w)α−1Γ(α)wldw  +M~rmax⁡{aα+l,bα+l}∫s1/s21(1−w)α−1Γ(α)wldw  +M~r[s1α+l∫01(1−w)α−1Γ(α)wldw       −s2α+l∫0s1/s2(1−w)α−1Γ(α)wldw] ≤|s1σ−α−l(1+s1)(1+s1μ)−s2σ−α−l(1+s2)(1+s2μ)|  ×max⁡{aα+l,bα+l}M~r∫01(1−w)α−1Γ(α)wldw  +M~rmax⁡{aα+l,bα+l}∫s1/s21(1−w)α−1Γ(α)wldw  +M~r|s1α+l−s2α+l|∫01(1−w)α−1Γ(α)wldw  +max⁡{aα+l,bα+l}∫s1/s21(1−w)α−1Γ(α)wldw ⟶0        uniformly  as  s1⟶s2  with  s1,s2∈[a,b]⊂(tk,tk+1].

So

(115) |s1σ−α−l(1+s1)(1+s1μ)(Tmx)(s1)−s2σ−α−l(1+s2)(1+s2μ)(Tmx)(s2)| =|s1σ−α−l(1+s1)(1+s1μ)   ×∫tks1(s1−s)α−1Γ(α)q(s)f(s,x(s),Dctk+px(s))ds   −s2σ−α−l(1+s2)(1+s2μ)   ×∫tks2(s2−s)α−1Γ(α)q(s)f(s,x(s),Dctk+px(s))ds|  +|x0||s1σ−α−l(1+s1)(1+s1μ)−s2σ−α−l(1+s2)(1+s2μ)|  +|s1σ−α−l(1+s1)(1+s1μ)−s2σ−α−l(1+s2)(1+s2μ)|  ×∑j=1k|Ij(tj,x(tj))|  +|s1σ−α−l(1+s1)(1+s1μ)−s2σ−α−l(1+s2)(1+s2μ)|  ×∑j=1k∫tk−1tk(tk−s)α−1Γ(α)|q(s)f(s,x(s),Dctk+px(s))|ds ≤|s1σ−α−l(1+s1)(1+s1μ)    ×∫0s1(s1−s)α−1Γ(α)q(s)f(s,x(s),Dc0+px(s))ds    −s2σ−α−l(1+s2)(1+s2μ)    ×∫0s2(s2−s)α−1Γ(α)q(s)f(s,x(s),Dc0+px(s))ds|   +|x0||s1σ−α−l(1+s1)(1+s1μ)−s2σ−α−l(1+s2)(1+s2μ)|   +|s1σ−α−l(1+s1)(1+s1μ)−s2σ−α−l(1+s2)(1+s2μ)|∑j=1∞Mrk   +M~r|s1σ−α−l(1+s1)(1+s1μ)−s2σ−α−l(1+s2)(1+s2μ)|   ×∑j=1ktkα+l∫01(1−w)α−1Γ(α)wldw ⟶0         uniformly  as  s1→s2  with  s1,s2∈[a,b]⊂(tk,tk+1].

It follows that

(116) |s1σ−α−l(1+s1)(1+s1μ)(Tmx)(s1) −s2σ−α−l(1+s2)(1+s2μ)(Tmx)(s2)|⟶0

uniformly as *s*_1_ → *s*_2_ with *s*_1_, *s*_2_ ∈ [*a*, *b*]⊂(*t*_*k*_, *t*_*k*+1_].

In a similar manner, one can find that

(117) |s1p+σ−α−l1+s1μ cDtk+p(Tmx)(s1)−s2p+σ−α−l1+s2μ cDtk+p(Tmx)(s2)| =|s1p+σ−α−l1+s1μ∫tks1(s1−s)α−p−1Γ(α−p)q(s)         ×f(s,x(s),Dctk+px(s))ds   −s2p+σ−α−l1+s2μ   ×∫tks2(s2−s)α−p−1Γ(α−p)q(s)   ×f(s,x(s),Dctk+px(s))ds| ⟶0         uniformly  as  s1⟶s2  with  s1,s2∈[a,b]⊂(tk,tk+1].

From (116) and (117), we deduce that *T*_*m*_ *W* is equicontinuous on finite closed interval on (*t*_*k*_, *t*_*k*+1_].

*Step 4.*   We prove that *T*_*m*_ *W* is equiconvergent as *t* → *t*_*k*_^+^  (*k* = 0,1, 2,…).

As in Lemma 10, *T*_*m*_ *W* is equiconvergent as *t* → 0^+^. For *t* → *t*_*k*_^+^, we have

(118) tσ−α−l(1+t)(1+tμ) ×|(Tmx)(t)−(∑j=1k∫tj−1tj(tj−s)α−1Γ(α)q(s)              ×f(s,x(s),Dctk+px(s))ds           +x0+∑j=1kIj(tj,x(tj)))| ≤tσ−α−l(1+t)(1+tμ)  ×∫tkt(t−s)α−1Γ(α)|q(s)f(s,x(s),Dctk+px(s))|ds ≤Mrtσ(1+t)(1+tμ)∫tk/t1(1−w)α−1Γ(α)wldw,tp+σ−α−l1+tμ|Dctk+(Tmx)(t)     −∫tkt(t−s)α−p−1Γ(α−p)q(s)       ×f(s,x(s)Dctk+x(s))ds|  ≤Mrtσ1+tμ∫tk/t1(1−w)α−p−1Γ(α−p)wldw.

Hence, *T*_*m*_ *W* is equiconvergent as *t* → *t*_*k*_^+^  (*k* = 1,2, 3,…).

*Step 5.* *T*_*m*_ *W* is equiconvergent as *t* → *∞*. Notice that

(119) tσ−α−l(1+t)(1+tμ) ×|(Tmx)(t)   −(x0+∑j=1∞Ij(tj,x(tj))      +∑j=1∞∫tj−1tj(tj−s)α−1Γ(α)q(s)          ×f(s,x(s),Dctk+px(s))ds)| ≤tσ−α−l(1+t)(1+tμ)  ×∫tkt(t−s)α−1Γ(α)|q(s)f(s,x(s),Dctk+px(s))|ds  +tσ−α−l(1+t)(1+tμ)  ×∑j=k+1∞∫tj−1tj(tj−s)α−1Γ(α)       ×|q(s)f(s,x(s),Dctk+px(s))|ds ≤M~rtσ(1+t)(1+tμ)∫tk/t1(1−w)α−1Γ(α)wldw   +M~rtσ−α−l(1+t)(1+tμ)   ×∑j=k+1∞tjα+l∫tj−1/tj1(1−w)α−1Γ(α)wldw ≤M~rMσ,μ11+tB(α,l+1)Γ(α)  +M~r1λ0μ−σ+1∑j=k+1∞1jμ−σ+1B(α,l+1)Γ(α) ⟶0 uniformly  in  W  as  t⟶∞  (k⟶∞),

(120) tp+σ−α−l1+tμ|Dctk+p(Tmx)(t)| ≤tp+σ−α−l1+tμtα+l−p  ×∫tk/t1(1−w)α−p−1Γ(α)M~rwldw ≤tσ1+tμ∫01(1−w)α−p−1Γ(α)M~rwldw ⟶0 uniformly  in  W  as  t⟶∞.

Hence, *T*_*m*_ *W* is equiconvergent as *t* → *∞*. This completes the proof in which *T*_*m*_ is completely continuous.

**Theorem 15**

Assume that (*H*_1_) and (*H*_2_) hold. Then IVP (7) has at least one solution *x* ∈ *X* if

(121) $$\begin{array}{c} \delta_{m}<1\;\;\;or\;\;\delta_{m}=1\;\;\;with\;\;N_{0}<1\;\;\;\text{or}\\ \delta_{m}>1\;with\;\;\frac{{||\Psi ||}^{1-\delta_{m}}{\left(\delta_{m}-1\right)}^{\delta_{m}-1}}{\delta_{m}^{\delta_{m}}}\geq N_{0}, \end{array}$$

where *N*_0_ = max⁡{*M*_2_, *M*_3_}, *M*_2_ is given by (83) and

(122) M3=∑i=1m[(Mσ,μB(α,l+1)Γ(α)    +1λ0μ−σ+1B(α,l+1)Γ(α)∑j=1∞1jμ−σ+1)   ×[Ai+Bi]   +Mσ−α−l,μ∑j=1∞Aji]||Ψ||δi−δm.

**Proof**

Let *Y* denote the Banach space equipped with the norm ||·|| (introduced in Section 2). Let *T*_*m*_ : *Y* → *Y* be an operator defined by (98). In view of Lemma 8, we need to show that the operator *T*_*m*_ has a fixed point in *Y* which will be a solution of IVP (7). By Lemma 14, *T*_*m*_ is well defined and completely continuous. Lets us introduce

(123) Φ(t)=C∫tkt(t−s)α−1Γ(α)q(s)ds+C∑j=1k∫tj−1tj(tj−s)α−1Γ(α)q(s)ds+x0+∑j=1kDj, t∈(tk,tk+1],  k=0,1,….

It is easy to show that Φ ∈ *Y*. Let $\overset{-}{r}>0$ and define

(124) $$\begin{array}{c} {\bar{\Omega }}_{\overset{-}{r}}=\left\{x\in Y:||x-\Phi ||\leq \overset{-}{r}\right\}. \end{array}$$

For $x\in {\bar{\Omega }}_{\overset{-}{r}}$ , we have $||x-\Phi ||\leq \overset{-}{r}$ . Then

(125) ||x||=max⁡{sup⁡t∈(0,∞)tσ−α−l(1+t)(1+tμ)|x(t)|,    sup⁡k=0,1,2,… sup⁡t∈(tk,tk+1]tp+σ−α−l1+tμ|Dctk+px(t)|}≤||x−Φ||+||Φ||≤r+||Φ||.

Using the assumptions (*H*_1_) and (*H*_2_), we find that

(126) tσ−α−l(1+t)(1+tμ)|(Tmx)(t)−Φ(t)| ≤tσ−α−l(1+t)(1+tμ)  ×∫tkt(t−s)α−1Γ(α)|q(s)|     ×|f(s,x(s),Dc0+px(s))−C|ds  +tσ−α−l(1+t)(1+tμ)  ×∑j=1k∫tj−1tj(tj−s)α−1Γ(α)|q(s)|      ×|f(s,x(s),Dc0+px(s))−C|ds  +tσ−α−l(1+t)(1+tμ)∑j=1k|Ij(tj,x(tj))−Dj| ≤tσ−α−l(1+t)(1+tμ)  ×∫tkt(t−s)α−1Γ(α)sl     ×[∑i=1mAi|tσ−α−l(1+t)(1+tμ)x(s)|δi       +∑i=1mBi|tp+σ−α−l1+tμ cD0+px(s)|δi]ds  +tσ−α−l(1+t)(1+tμ)  ×∑j=1k∫tj−1tj(tj−s)α−1Γ(α)sl      ×[∑i=1mAi|tσ−α−l(1+t)(1+tμ)x(s)|δi         +∑i=1mBi|tp+σ−α−l1+tμ cD0+px(s)|δi]ds  +tσ−α−l(1+t)(1+tμ)  ×∑j=1k∑i=1mAji|tjσ−α−l(1+tj)(1+tjμ)x(tj)|δi ≤tσ−α−l(1+t)(1+tμ)∫tkt(t−s)α−1Γ(α)slds  ×∑i=1m[Ai+Bi]||x||δi  +tσ−α−l(1+t)(1+tμ)∑j=1k∫tj−1tj(tj−s)α−1Γ(α)slds  ×∑i=1m[Ai+Bi]||x||δi  +tσ−α−l(1+t)(1+tμ)∑j=1k ∑i=1mAji||x||δi ≤tσ(1+t)(1+tμ)∫tk/t1(1−w)α−1Γ(α)wldw  ×∑i=1m[Ai+Bi]||x||δi  +tσ−α−l(1+t)(1+tμ)  ×∑j=1ktjα+l∫tj−1/tj1(1−w)α−1Γ(α)wldw  ×∑i=1m[Ai+Bi]||x||δi  +Mσ−α−l,μ∑i=1m ∑j=1∞Aji||x||δi ≤Mσ,μ∫01(1−w)α−1Γ(α)wldw  ×∑i=1m[Ai+Bi]||x||δi  +tσ−α−ltμ+1∑j=1ktjα+l∫tj−1/tj1(1−w)α−1Γ(α)wldw  ×∑i=1m[Ai+Bi]||x||δi  +Mσ−α−l,μ∑i=1m ∑j=1∞Aji||x||δi ≤Mσ,μ∫01(1−w)α−1Γ(α)wldw  ×∑i=1m[Ai+Bi]||x||δi  +∑j=1k1tjμ−σ+1∫01(1−w)α−1Γ(α)wldw  ×∑i=1m[Ai+Bi]||x||δi  +Mσ−α−l,μ∑i=1m ∑j=1∞Aji||x||δi ≤Mσ,μB(α,l+1)Γ(α)∑i=1m[Ai+Bi]||x||δi  +∑j=1∞1jμ−σ+1λ0μ−σ+1B(α,l+1)Γ(α)  ×∑i=1m[Ai+Bi]||x||δi  +Mσ−α−l,μ∑i=1m ∑j=1∞Aji||x||δi =[(Mσ,μB(α,l+1)Γ(α)+1λ0μ−σ+1B(α,l+1)Γ(α)∑j=1∞1jμ−σ+1)   ×∑i=1m[Ai+Bi]+Mσ−α−l,μ∑i=1m ∑j=1∞Aji]  ×[r+||Φ||]δi ≤N1[r+||Φ||]δm.

Furthermore, we have

(127) tp+σ−α−l1+tμ|Dctk+p(Tmx)(t)−Dctk+pΦ(t)| ≤tp+σ−α−l1+tμ  ×∫tkt(t−s)α−p−1Γ(α−p)|q(s)|     ×|f(s,x(s),Dctk+px(s))−C|ds ≤tp+σ−α−l1+tμ  ×∫tkt(t−s)α−p−1Γ(α−p)sl  ×[∑i=1mAi|tσ−α−l(1+t)(1+tμ)x(s)|δi    +∑i=1mBi|tp+σ−α−l1+tμ cDtk+px(s)|δi]ds ≤tp+σ−α−l1+tμ  ×∫0t(t−s)α−p−1Γ(α−p)sl    ×[∑i=1mAi||x||δi+∑i=1mBi||x||δi]ds ≤Mσ,μB(α−p,l+1)Γ(α−p)  ×[∑i=1mAi||x||δi+∑i=1mBi||x||δi] ≤Mσ,μB(α−p,l+1)Γ(α−p)∑i=1m[Ai+Bi]||x||δi ≤Mσ,μB(α−p,l+1)Γ(α−p) ∑i=1m[Ai+Bi][r+||Φ||]δi ≤[r+||Φ||]δmN2.

Thus, it follows that

(128) $$\begin{array}{c} ||T_{m}x-\Phi ||\leq {\left[r+||\Phi ||\right]}^{\delta_{m}}N_{0}. \end{array}$$

Now we discuss the cases for different values of *δ*_*m*_.

(i) For *δ*_*m*_ < 1, we can choose ${\overset{-}{r}}_{0}>0$ sufficiently large so that $[{\overset{-}{r}}_{0}+{||\Phi ||]}^{\delta_{m}}N_{0}<{\overset{-}{r}}_{0}$ . Let $\Omega_{{\overset{-}{r}}_{0}}=\{x\in Y:||x||<{\overset{-}{r}}_{0}\}$ . It is easy to show that $T_{m}{\bar{\Omega }}_{{\overset{-}{r}}_{0}}\subset {\bar{\Omega }}_{{\overset{-}{r}}_{0}}$ . Then, the Schauder fixed point theorem implies that the operator *T*_*m*_ has a fixed point $x\in {\bar{\Omega }}_{{\overset{-}{r}}_{0}}$ , which is a bounded solution of IVP (7).

(ii) For *δ*_*m*_ = 1, we select

(129) $$\begin{array}{c} {\overset{-}{r}}_{0}\geq \frac{||\Psi ||N_{0}}{1-N_{0}}. \end{array}$$

Let $\Omega_{{\overset{-}{r}}_{0}}=\{x\in Y:||x||<{\overset{-}{r}}_{0}\}$ . It can easily be shown that $T_{m}{\bar{\Omega }}_{{\overset{-}{r}}_{0}}\subset {\bar{\Omega }}_{{\overset{-}{r}}_{0}}$ . Then, the Schauder fixed point theorem applies and the operator *T*_*m*_ has a fixed point $x\in {\bar{\Omega }}_{{\overset{-}{r}}_{0}}$ , which is a bounded solution of IVP (7).

(iii) For *δ*_*m*_ > 1, we set $\overset{-}{r}={\overset{-}{r}}_{0}=||\Phi ||/\left(\delta_{m}-1\right)$ so that

(130) $$\begin{array}{c} \frac{{\overset{-}{r}}_{0}}{{\left({\overset{-}{r}}_{0}+||\Phi ||\right)}^{\delta_{m}}}=\frac{{||\Phi ||}^{1-\delta_{m}}{\left(\delta_{m}-1\right)}^{\delta_{m}-1}}{\delta_{m}^{\delta_{m}}}\geq N_{0}. \end{array}$$

Let $\Omega_{{\overset{-}{r}}_{0}}=\{x\in Y:||x||<{\overset{-}{r}}_{0}\}$ . Then we can show that $T_{m}{\bar{\Omega }}_{r_{0}}\subset {\bar{\Omega }}_{r_{0}}$ . Thus, by the Schauder fixed point theorem, the operator *T*_*m*_ has a fixed point $x\in {\bar{\Omega }}_{{\overset{-}{r}}_{0}}$ , which is a solution of IVP (7). This completes the proof.

**Theorem 16**

Suppose that (*H*_1_) and (*H*_2_) hold with *δ*_*m*_ = 1. Then IVP (7) has a unique solution *x* ∈ *Y* if *N*_0_ < 1.

**Proof**

The proof is similar to that of Theorem 12, so we omit it.

## 5. Applications

Malthusian geometrical law is expressed as *N*′(*t*) = *rN*(*t*), where *N*(*t*) is the population at time *t* and *r* is the proportionality constant. When the growth of the population in any environment is stopped due to the density of the population, this model modifies to a nonlinear logistic model of the form *N*′(*t*) = *rN*(*t*)(1 − *N*(*t*)/*π*). The generalization of the nonlinear logistic model is represented by *N*′(*t*) = *rN*(*t*)[1 − (*N*(*t*)/*π*)^*α*^]/*α*. For *α* → 0, the model is known as the Gompertz model and can be found in the literature on actuarial science and mortality analysis of elderly person [31].

In [32], Das et al. presented the following fractional-order logistic model (Das Model):

(131) $$\begin{array}{c} D_{0^{+}}^{\beta }N\left(t\right)=\frac{r}{\alpha }N\left(t\right)\left[1-{\left(\frac{N(t)}{\pi }\right)}^{\alpha }\right],\;0<\beta \leq 1. \end{array}$$

In [33], the authors presented the following logistic model with fractional order:

(132) $$\begin{array}{c} {}^{c}D_{0^{+}}^{\alpha }x\left(t\right)=x\left(t\right)\left[a\left(t\right)-b\left(t\right)\left(x\left(t\right)\right)\right],\;t\in \left(0,\infty \right),\;\;t\neq t_{k},\\ \Delta x\left(t_{k}\right)=I_{k}\left(x\left(t_{k}^{-}\right)\right),\;k=1,2,\ldots ,\\ x\left(0\right)=x_{0}, \end{array}$$

where *T* > 0 is a constant, *I*_*k*_ : *R* → *R*  (*k* = 1,2,…, *m*) are impulse functions, *a*(*t*)∈[*a*_∗_, a*], and *b*(*t*)∈[*b*_∗_, b*] with *a*_∗_ > 0, *b*_∗_ > 0.

As an application of the main results established in the paper, we discuss the sufficient conditions for the existence and asymptotic behavior of solutions for the logistic models:

(133) Dc0+αx(t)=x(t)[a(t)−b(t)(x(t))δ], t∈(0,∞),  t≠tk,Δx(tk)=Ik(tk,x(tk)), k=1,2,…,x(0)=x0,

(134) Dc∗αx(t)=x(t)[a(t)−b(t)(x(t))δ], t∈(0,∞),  t≠tk,Δx(tk)=Ik(tk,x(tk)), k=1,2,…,x(0)=x0,

where 0 < *t*_1_ < *t*_2_ < *t*_3_ < ⋯, *α* ∈ (0,1], *δ* > 0, *a*, *b* : (0, *∞*) → *R* are continuous functions, and *a*_*k*_ ∈ *R* → *R*  (*k* = 1,2, 3,…) are constants.

**Theorem 17**

Suppose that

(135) (1+t)(1+tμ)tσ−α−la(t)≤a0,((1+t)(1+tμ)tσ−α−l)δ+1b(t)≤b0,           t∈(0,∞),

and there exists *D*_*k*_ ∈ *R*, *A*_*k*1_, *A*_*k*2_ ≥ 0 such that

(136) |Ik(tk,(1+t)(1+tμ)tσ−α−lu)−Dk| ≤Ak1|u|+Ak2|u|2, k=1,2,3,…,  u∈R.

Then IVP (133) has at least one solution if

(137) $$\begin{array}{c} 4||\Phi ||M_{0}\leq 1, \end{array}$$

where

(138) Φ(t)=x0+∑j=1kDj, t∈(tk,tk+1],  k=0,1,2,…,M1=[Mσ,μB(α,l+1)Γ(α)a0+Mσ−α−l,μ∑j=1∞Aj1]||Ψ||−1+Mσ,μB(α,l+1)Γ(α)b0+Mσ−α−l,μ∑j=1∞Aj2,M2=Mσ,μB(α−p,l+1)Γ(α−p)(a0+b0),M0=max⁡{M1,M2}.

**Proof**

Let *f*(*t*, *u*, *v*) = *u*[*a*(*t*) − *b*(*t*)*u* ^*δ*^]. Then

(139) |f(t,(1+t)(1+tμ)tσ−α−lu,1+tμtp+σ−α−l)| =(1+t)(1+tμ)tσ−α−la(t)|u|  +b(t)((1+t)(1+tμ)tσ−α−l)δ+1|u|δ+1 ≤a0|u|+b0|u|δ+1.

In association with Theorem 11, we choose *C* = 0, *A*_1_ = *a*_0_, *A*_1_ = *a*_1_, *δ*_1_ = 1, *δ*_2_ = 2, *B*_1_ = *B*_2_ = 0. Then the conditions (*H*_1_) and (*H*_2_) hold. By Theorem 11, IVP (133) has at least one solution. This completes the proof.

**Theorem 18**

Suppose that

(140) (1+t)(1+tμ)tσ−α−la(t)≤a0,((1+t)(1+tμ)tσ−α−l)δ+1b(t)≤b0,           t∈(0,∞)

and there exists *D*_*k*_ ∈ *R*, *A*_*k*1_, *A*_*k*2_ ≥ 0 such that

(141) |Ik(tk,(1+t)(1+tμ)tσ−α−lu)−Dk| ≤Ak1|u|+Ak2|u|2, k=1,2,3,…,  u∈R.

Then IVP (134) has at least one solution if

(142) $$\begin{array}{c} 4N_{0}||\Psi ||\leq 1, \end{array}$$

where

(143) Ψ(t)=x0+∑j=1kDj, t∈(tk,tk+1],  k=0,1,2,…,M3=[(Mσ,μB(α,ł+1)Γ(α)   +1λ0μ−σ+1B(α,l+1)Γ(α)∑j=1∞1jμ−σ+1)a0  +Mσ−α−l,μ∑j=1∞Aj1]||Ψ||−1 +(Mσ,μB(α,l+1)Γ(α)    +1λ0μ−σ+1B(α,l+1)Γ(α)∑j=1∞1jμ−σ+1)b0+Mσ−α−l,μ∑j=1∞Aj2,M2=Mσ,μB(α−p,l+1)Γ(α−p)(a0+b0),N0=max⁡{M2,M3}.

**Proof**

The proof immediately follows from Theorem 15.
